# Two types of soybean diacylglycerol acyltransferases are differentially involved in triacylglycerol biosynthesis and response to environmental stresses and hormones

**DOI:** 10.1038/srep28541

**Published:** 2016-06-27

**Authors:** BeiBei Chen, Junejie Wang, Gaoyang Zhang, Jiaqi Liu, Sehrish Manan, Honghong Hu, Jian Zhao

**Affiliations:** 1 National Key Laboratory of Crop Genetic Improvement, College of Plant Science & Technology, Huazhong Agricultural University, Wuhan, 430070, China; 2 College of Agronomy, Jilin Agricultural University, Changchun, 130047, China; 3 National Key Laboratory of Crop Genetic Improvement, College of Life Science & Technology, Huazhong Agricultural University, Wuhan, 430070, China

## Abstract

Diacylglycerol acyltransferases (DGATs) play a key role in plant triacylglycerol (TAG) biosynthesis. Two type 1 and 2 DGATs from soybean were characterized for their functions in TAG biosynthesis and physiological roles. *GmDGAT1A* is highly expressed in seeds while *GmDGAT2D* is mainly expressed in flower tissues. They showed different expression patterns in response to biotic and abiotic stresses. *GmDGAT2D* was up-regulated by cold and heat stress and ABA signaling, and repressed by insect biting and jasmonate, whereas *GmDGAT1A* show fewer responses. Both GmDGAT1A and GmDGAT2D were localized to the endoplasmic reticulum and complemented the TAG deficiency of a yeast mutant H1246. *GmDGAT2D*-transgenic hairy roots synthesized more 18:2- or 18:1-TAG, whereas *GmDGAT1A* prefers to use 18:3-acyl CoA for TAG synthesis. Overexpression of both *GmDGAT*s in Arabidopsis seeds enhanced the TAG production; *GmDGAT2D* promoted 18:2-TAG in wild-type but enhanced 18:1-TAG production in *rod1* mutant seeds, with a decreased 18:3-TAG. However, GmDGAT1A enhanced 18:3-TAG and reduced 20:1-TAG contents. The different substrate preferences of two DGATs may confer diverse fatty acid profiles in soybean oils. While GmDGAT1A may play a role in usual seed TAG production and GmDGAT2D is also involved in usual TAG biosynthesis in other tissues in responses to environmental and hormonal cues.

Soybean [*Glycine max* (L.) Merr.] is the largest oilseed crop in the world, providing almost 28% edible vegetable oils for human^1^,^2^. However, compared to rapeseeds and sunflower seeds that usually contain around 40% oils, there might be a large space to improve the oil content in soybean seeds^1^,^2^. The limited understanding of soybean oil biosynthesis has become a major obstacle for the improvement of soybean oil production^3^. In higher plants triacylglycerols (TAGs) are the most common storage lipids in seed as the form of lipid droplets. The biosynthesis of TAGs is not restricted to seeds, but widely occurs in most vegetative tissues, although their exact function remains to be determined^2^,^4^,^5^. However, an increasing body of evidence suggests TAG and biosynthetic genes participate in plant stress or defense response^2^,^4^,^5^,^6^. The plastid is the major place where the *de novo* biosynthesis of fatty acids (FAs) occurs; then these FAs are transported out of plastids in active forms into the cytosol, where fatty acyl-CoAs form precursor pools. Fatty acyl-CoAs and glycerol 3-phosphate are transported into the endoplasmic reticulum (ER) to synthsize TAGs and then assembled into oil bodies^2^,^5^,^7^. The synthesis of phospholipids and TAGs in ER contains various acyltransferases, which have substrate specificities towards fatty acyl-CoAs as donors and different glycerol backbones as acceptors. Thus acyltransferases not only conduct the acyl editing on DAG and eventual product TAGs in the ER, but also manipulation of these acyltransferases leads to increased TAG production in plants^7^.

Diacylglycerol acyltransferases (DGATs) catalyze the esterification of the acyl group of acyl-CoAs at the sn-3 position of sn-1, 2-diacylglycerol (DAG) molecules, the final step in the Kennedy pathway leading to TAG production. DGATs play essential roles in determining the quality and quantity of acyl-CoA flux into TAG synthesis through Kennedy pathway. At least four classes of DGATs have been identified based on structure and activity^7^. The most common is type 1 (DGAT1), which contributes to the synthesis of the majority of TAGs^7^. DGAT1 is usually predicted to possess six or more transmembrane domains and belongs to the membrane-bound O-acyltransferase (MBOAT) family^8^. Studies have confirmed that DGAT1 affects the TAG levels in seeds of many species^9^,^10^,^11^,^12^,^13^. DGAT2 proteins possess only two to three predicted transmembrane domains and belongs to the monoacylgycerol acyltransferase (MGAT) family. A third type of DGATs is similar to DGAT2 with both wax ester synthase (WS) and DGAT activity, including ADP1 from *Acinetobacter calcoaceticus*^14^ and ADP1 homologs from Arabidopsis and petunia (*Petunia hybrida*)^15^,^16^. A fourth type, a soluble cytosolic DGAT (DGAT3), was identified in peanut (*Arachis hypogaea*) and Arabidopsis^17^,^18^.

DGAT1s from different plants, such as Arabidopsis, have been shown to have major contribution to TAG synthesis, whereas the role of DGAT2s from these plants is not fully understood^19^,^20^. Arabidopsis DGAT2 has recently been reported for a much weaker function in TAG synthesis compared with DGAT1^16^,^21^,^22^,^23^. However, the mostly characterized plant DGAT2s are active for synthesis of unusual TAG using hydroxyl, conjugated or epoxy FAs. For examples, DGAT2 in tung tree (*Vernicia fordii*) uses eleostearic acid, CpDGAT2 in *Claviceps purpurea* and RcDGAT2 in caster bean (*Ricinus communis*) use ricinoleate, and VgDGAT in *Vernonia galamensis* uses vernolic acid for production of unusual TAGs, including trieleostearin, triricinolein, or epoxyl-TAG^10^,^24^,^25^,^26^. DAGT2s from fungi and animals were shown active for TAG synthesis^25^,^27^,^28^,^29^,^30^. Thus, knowledge about the redundant functions of DGAT1 and 2s from plants that do not synthesize hydroxy TAGs, such as Arabidopsis and soybean is still very limited^20^. In particular, tetraploid Soybean genome contains at least 10 putative DGAT genes: 3 *DGAT1s*, 5 *DGAT2s*, and 2 *DGAT3s*^16^,^31^,^32^ (Supplementary Fig. S1). GmDGAT1A and GmDGAT1B are active in TAG synthesis in yeast and their transcripts are abundant in developing seeds and associated with maximal TAG accumulation^16^. GmDGAT2C played role in seed yield, whereas GmDGAT1B was associated with seed yield and protein concentration^32^. Since soybean seeds do not produce unusual oil but with such abundant copies of DGATs, it is of great interest and needs to be understood their functional redundancy, conservation, and divergence. Here, we report the functional comparison study on type 1 and 2 soybean diacylglycerol acyltransferases, GmDGAT1A and GmDGAT2D. The expression of both *GmDGAT1A* and *GmDGAT2D* restored the *in vivo* synthesis of TAG in yeast strain *Saccharomyces cerevisiae* H1246. *GmDGAT2D* expression in soybean hairy roots and Arabidopsis lead to similar function in regular TAG production like fungal and animal DGAT2, but differ from other known plant DGAT2s, which synthesize unusual TAGs with hydroxy FAs. Our study also suggests that *GmDGAT1A* and *2D* are differentially regulated by jasmonate during insect and wounding responses and ABA for cold and heat stress response, indicating their different functions in soybean stress responses.

## Results

### Identification of diacylglycerol acyltransferase 1A and 2D from *Glycine max*

To understand the functions of DGATs in biosynthesis of soybean oils that do not contain unusual TAGs, such as hydroxyl TAGs, we cloned *GmDGAT1A* and *GmDGAT2D*. Homology searches revealed GmDGAT2D shares highest identity with other plant DGAT2 genes from *Glycine soja* and *Phaseolus vulgaris*. GmDGAT2D shares 68% identity and 85% similarity with OeDGAT2 from olive (*Olea europaea*), 68% identity and 83% similarity with AtDGAT2, 67% identity and 81% similarity with HaDGAT2 from sunflower (*Helianthus annuus*), and 64% identity and 80% similarity with *Ricinus communis* RcDGAT2 (Supplementary Fig. S1). GmDGAT1A has nine predicted transmembrane (TM) domains whereas GmDGAT2D has only two TM domains, with a short head and a long tail inside the ER, and a cytosolic motif between TMs (Supplementary Fig. S1). These features are similar to fungal DGAT2s^10^,^20^,^33^.

**Figure 1:**
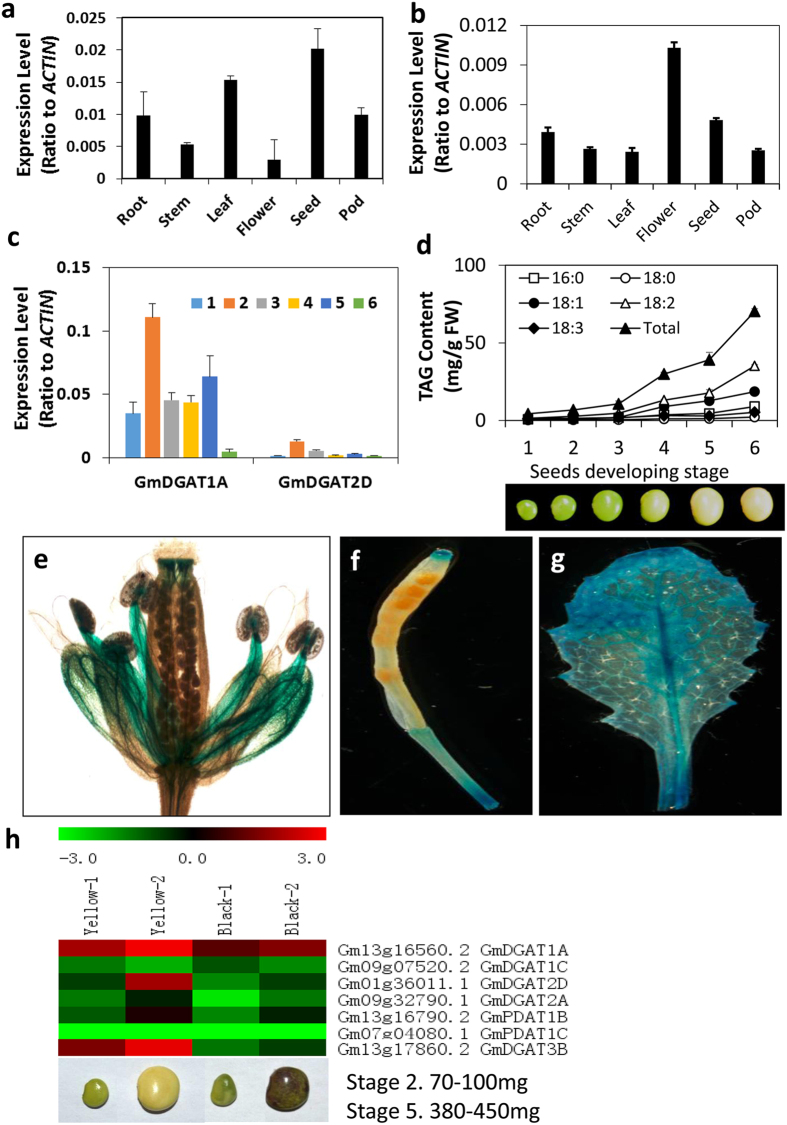
*GmDGAT1A* and *GmDGAT2D* expression pattern. The tissue expression patterns of *GmDGAT1A* and *GmDGAT2D* in nodule, root, stem, leaf, flower, pod, seed was tested by qRT-PCR with actin as internal standard. Seeds at different development stages were harvested. Six developmental stages are: stage 1. 40–70 mg; stage 2. 80–100 mg; stage 3.150–200 mg; stage 4. 250–300 mg; stage 5. 350–430 mg; stage 6. 320–350 mg. **(a)** Tissue specific expression of *GmDGAT2D.* **(b)** Tissue specific expression of *GmDGAT1A.* **(c)** *GmDGAT1A* and *GmDGAT2D* expression at various seed developmental stages. **(d)** TAG accumulation and fatty acid composition changes in various seed developmental stages **(e–g)** *GmDGAT2D* promoter-driven *GUS* expression in Arabidopsis flower **(e),** *GmDGAT2D* promoter-driven *GUS* expression in Arabidopsis silique (**f**) and leaf (**g**). **(h)** *GmDGAT2D* and *GmDGAT1A* expression patterns in comparison of other DAGT genes. Soybean Illumina expression data obtained from developing seeds of two varieties with PI 84970 (Hokkaido, seed with a black seed coat) and PI 518671 (Williams 82, seed with yellow seed coat) at the developmental stages of 2 and 5. Data are presented as averages of three biological replicates ± SD.

Quantitative RT-PCR (qRT-PCR) data indicated that *GmDGAT1A* is mainly expressed in seeds, but also in tissues like roots, leaves, flowers, at relatively lower levels. *GmDGAT2D* transcripts were high in flowers and seeds, but also present in other tissues at lower levels, such as roots, leaves and pods (Fig. 1a, Supplementary Fig. S2). The transcripts of *GmDGAT1A* and *2D* at soybean developing seeds were also examined (Fig. 1c). Both expression patterns of *GmDGAT1A* and *2D* are consistent with oil accumulation (Fig. 1d, Supplementary Fig. S2). The patterns seem also consistent with our transcriptomic data from soybean seeds at two developmental stages. The transcriptomic data showed that both *GmDGAT1A* and *2D* transcripts increased with development, and were higher in yellow seeds than in black seeds, which is in line with higher oil contents in yellow seeds than in black seeds (Fig. 1h). These results suggest that GmDGAT1A and 2D may play role in seed oil biosynthesis.

Analysis of *GUS* expression driven by *GmDGAT2D* promoter (pro*GmDGAT2D::GUS*) in transformed Arabidopsis suggested that the pro*GmDGAT2D::GUS* is mainly expressed in flower tissues, such as anther filament, stigma and petals. The GUS activity in leaf vascular tissues was also observed (Fig. 1e,g). Its expression in immature seeds was not observed. These results indicate that *GmDGAT2D* expression in non-seed tissues is relatively higher.

**Figure 2:**
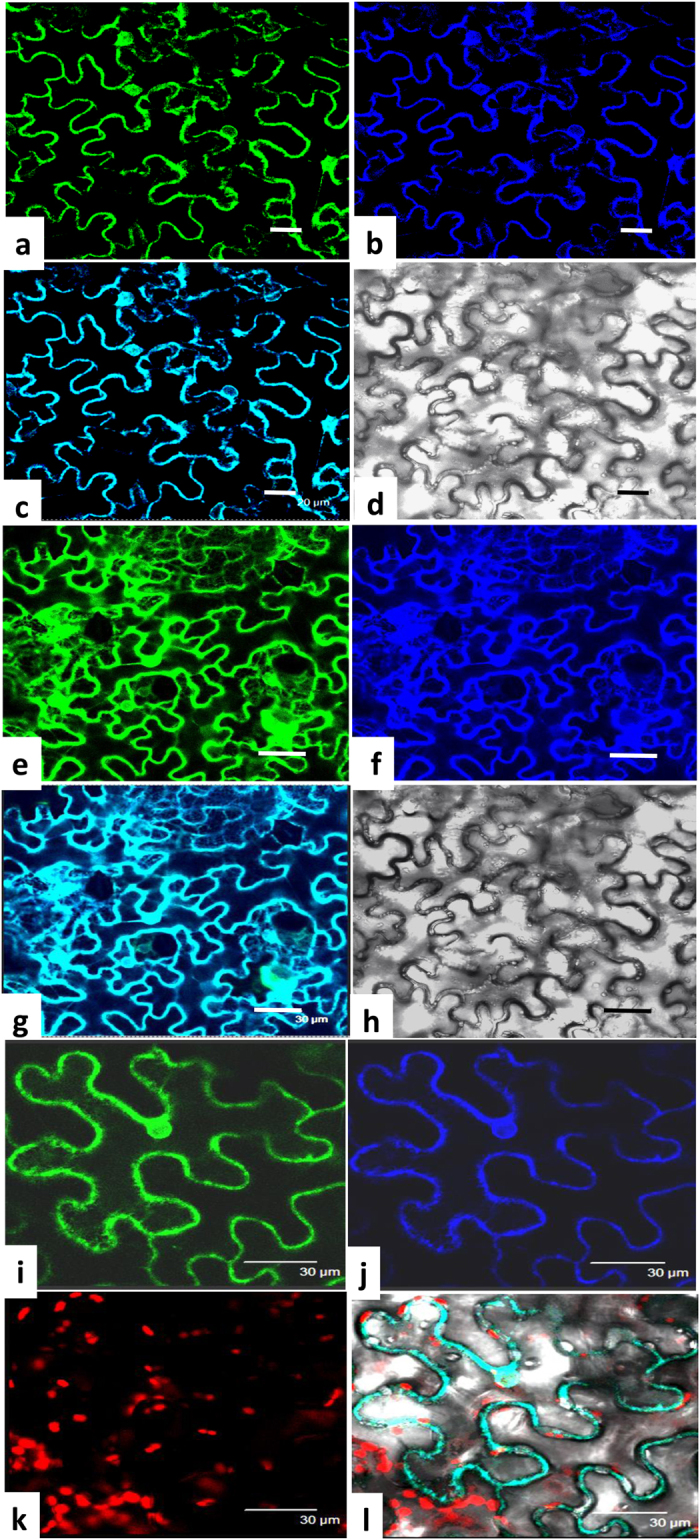
Subcellular localization of GmDGAT-1A and -2D. *GmDGAT1A-GFP*, *GmDGAT2D-GFP,* and a ER marker *CD3-959-mCherry*, driven by the cauliflower mosaic virus 35S promoter, were transiently expressed in tobacco leaf epidermal cells. Materials were viewed by confocal microscopy. Represatative photos were shown. **(a–d)** Localization of GmDGAT1A-GFP. Image of GmDGAT1A-GFP (**a**), the blue fluorescence image of ER marker CD3-959-mCherry (**b**), and the merged image of GmDGAT1A-GFP and CD3-959-mCherry (**c**), and the bright field image (**d**). Bars = 20 μm. **(e–h)** Localization of GmDGAT2D-GFP. Image of GmDGAT2D-GFP (**e**), image of ER marker CD3-959-mCherry (**f**), the merged image of GmDGAT2D-GFP and ER marker CD3-959-mCherry (**g**), and the bright field image of cells (**h**). Bars = 30 μm. **(i–l)** Enlarged image of GmDGAT2D-GFP (**i**), image of ER marker (**j**), the chloroplast autofluorescence images (**k**), and merged image of GmDGAT2D-GFP, ER marker, and chloroplast autofluorescence in a bright field (**l**). Bars = 30 μm.

To further understand the functions of GmDGAT1A and 2D, we examined their subcellular localization. GmDGAT2D has two TM domains and GmDGAT1A with nine TM domains (Supplementary Fig. S1). GmDGAT1A - and 2D-green fluorescent protein (GFP) was co-localized with the CD3-959-mCherry, a well-known endoplasmic reticulum (ER) marker (Fig. 2a–h)^33^. The distribution of GmDGAT2D-GFP fusion showed overlapping patterns with the ER. Thus two GmDGATs were primarily associated with the ER, as further referenced by chloroplast autoflourescence (Fig. 2i–l).

### GmDGAT1A and GmDGAT2D are functional DGATs when expressed in yeast

**Figure 3:**
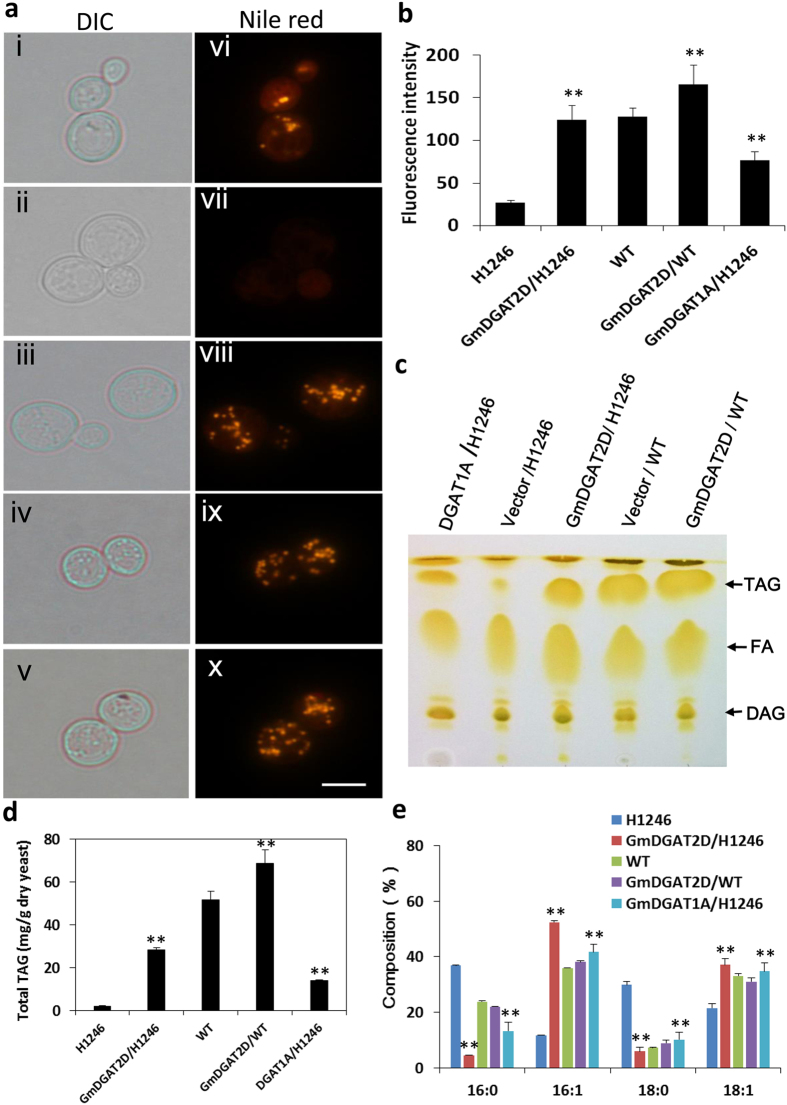
Functional expression of *GmDGATs* in yeast cells. The quadruple mutant *Saccharomyces cerevisiae* strain H1246 and the wild-type strain YPH499a were transformed with *GmDGAT* genes and vector control. **(a)** Nile red staining of oil drops in *GmDGAT1A* (i, vi)*-* and *2D (*iii, viii)- or pYESDEST52 vector (ii, vii)-expressing yeast mutant strain H1246, and vector (iv, ix) and *GmDGAT2D* (v, x expressed in wild-type strain YPH499a cells. TAG was extracted and separated by thin-layer chromatography and TAG spots were scraped for analyzing compositions of fatty acids by GC. **(b)** Nile Red Assay on oil drops and fluorescence-based TAG content. **(c)** Thin-layer chromatography of lipids extracted from yeast transformants. DAG, fatty acids (FAs), and TAG spots were labeled out. TAG spots were scraped off from the thin-layer chromatography (TLC) plates and were extracted for analysis of TAG contents and compositions with GC. **(d)** Total TAG production in yeast mutant strain H1246 and wild-type strain YPH499a, Total TAG was measured by GC. **(e)** Fatty acid compositions of TAG produced in *GmDGAT2D*- or *pYESDEST52* empty vector-expressing yeast mutant strain H1246 and wild-type strain YPH499a. All data are presented by mean of at least three biological replicates ± SD. *P < 0.05 and **P < 0.01 by Student’s *t* test for significant difference.

The ORFs of *GmDGAT1A* and *GmDGAT2D* were expressed in the TAG-deficient yeast (*Saccharomyces cerevisiae*) strain H1246, in which all four acyltransferase genes contributing to TAG synthesis were deleted^34^. When the *GmDGAT1A* and *GmDGAT2D* driven by a galactose-inducible GAL10 promoter in the vector pYESDEST52 were expressed in H1246 cells, many large and distinct oil bodies were observed in the Nile red-stained H1246 mutant cells after 24 h of galactose induction (Fig. 3a). Nile red has been broadly used for detecting TAG levels in numerous organisms, and used as a proper indicator of DGAT activity in H1246 strain^35^. As expected, no fluorescence was observed in H1246 yeast cells transformed with empty vector. Furthermore, overexpression of *GmDGAT2D* in a wild-type yeast strain also showed enhanced density and size of intracellular oil bodies (Fig. 3a). Quantification of Nile red fluorescence intensity of these cells suggested that, the fluorescence intensity was very low in the control H1246 cells, but double in *GmDGATs*–expressing H1246 cells (Fig. 3b). Furthermore, *GmDGAT1A- and 2D*-expressing wild-type cells also showed a stronger intensity than the empty vector-expressing wild-type cells. Nile red has been broadly used for detecting TAG levels in numerous organisms, and also used as a proper indicator of DGAT activity in H1246 strain^35^. TLC analysis of extracted lipids from these *GmDGAT*-expressing cells revealed a strong accumulation of TAG that was almost absent from H1246 cells only expressing empty vector (Fig. 3c). The strong FA bands shown on preparative TLC plates may come from hydrolysis of lipids during extraction process, rather than endogenous fatty acids^34^. Further analysis of the contents and compositions of TAGs separated by TLC confirmed that the total TAGs increased in *GmDGAT1A-* and *2D*-expressing yeast cells (Fig. 3d). Both GmDGAT1A and 2D showed DGAT activity sufficient to restore TAG synthesis in H1246 strain. The endogenous FA profile of H1246 consists mainly of palmitic acid (16:0), palmitoleic acid (16:1), stearic acid (18:0), and oleic acid (18:1). *GmDGAT1A* and *2D* expression triggered an increase in TAGs with 16:1 and 18:1 fatty acyl chains, but decreases in 16:0 and 18:0 fatty acyl chains (Fig. 3e), suggesting that both GmDGAT1A and 2D preferred to utilize 16:1 and 18:1 acyl-CoAs to synthesize TAGs in yeast. Overexpression of *GmDGAT2D* in wild-type yeast strain slightly increased 16:1/16:0 ratios.

### Ectopic expression of *GmDGATs* in soybean hairy roots promotes TAG accumulation

**Figure 4:**
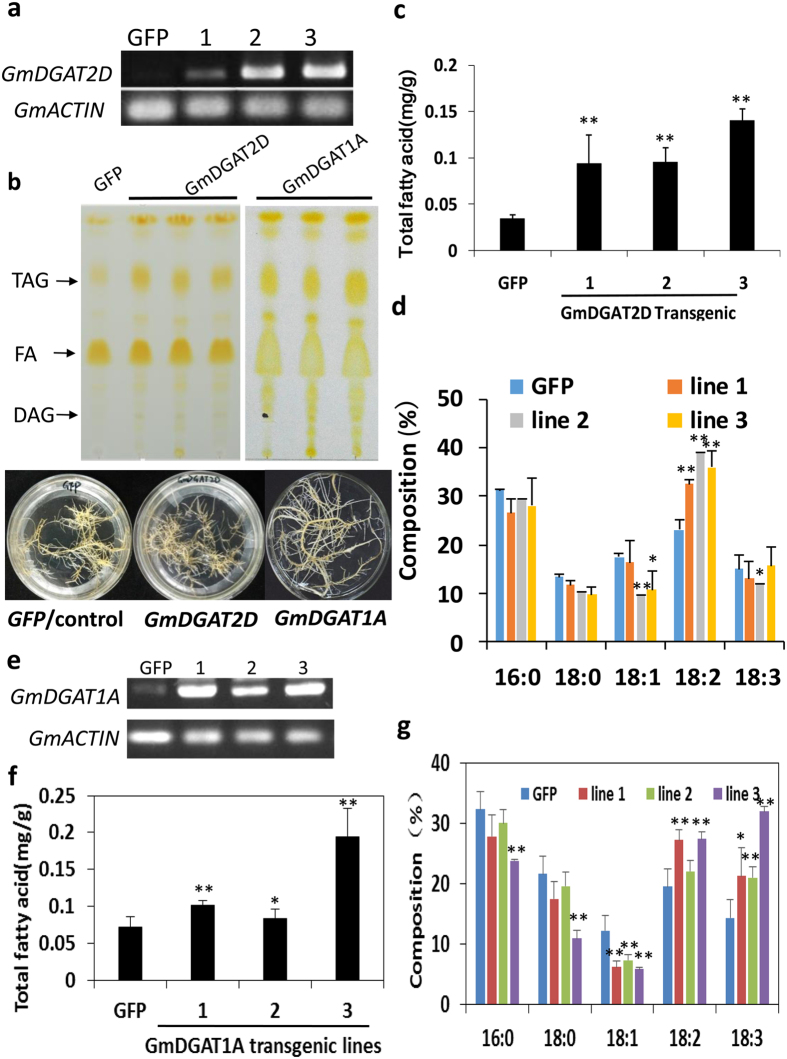
Ectopic expression of *GmDGATs* in soybean hairy roots. The cotyledons of germinating soybean seeds were used for infection by Agrobacteria K599 harboring *pB2GW7-GmDGAT2D* or –*GmDGAT1A*. Generated transgenic hairy roots selected on ppt-medium were confirmed by qRT-PCR for target gene expression, and analyzed for TAG production, as compared with GFP-expressing hairy roots as control. Total neutral lipids were extracted and separated by TLC. TAG spots were scraped off from TLC plates and extracted for determination of total contents and compositions by using GC. **(a)** qRT-PCR confirmation of *GmDGAT2D* expression in soybean transgenic hairy roots. **(b)** TLC analysis of neutral lipids extracted from hairy root transformants. DAG, fatty acids (FAs), and TAG spots were labeled out. Bottom panel shows the photos of soybean transgenic hairy roots. Hairy roots expressing free GFP vector were used as a control. **(c)** Quantification of total TAGs from hairy roots ectopically expressing *GmDGAT2D* compared with *GFP* control. **(d)** Fatty acid composition of TAGs from hairy roots expressing *GmDGAT2D* and *GFP* control. **(e)** qRT-PCR confirmation of *GmDGAT1A* expression in soybean transgenic hairy roots. **(f)** Total TAGs in *GmDGAT1A*-expressing hairy roots, GFP-hairy roots are used as a control. **(g)** Fatty acid composition of TAGs from hairy roots expressing *GmDGAT1A* and *GFP* control. All data are expressed as mean ± SD (n > 4); three lines are representative of more than 10 transgenic hairy root lines were analyzed. *P < 0.05 and **P < 0.01 by Student’s *t* test for significant difference.

To further understand the roles of GmDGAT1A and 2D in oil production, we expressed *GmDGAT1A* and *2D,* driven by a CaMV 35S promoter, in hairy roots derived from soybean cotyledons, which has been used as an efficient approach to verify functions of soybean genes^36^, giving that the lower transformation rate and time-consuming of soybean plant transformation. Endogenous expression of *GmDGAT1A* and *2D* in hairy roots is low, and TAG contents in these hairy roots were also much lower than in soybean seeds. The ectopic expression of *GmDGAT1A* and *2D* was confirmed with semi-quantitative RT-PCR and qRT-PCR; and TAG contents were confirmed with TLC analysis in combination with gas chromatography (GC) measurement (Fig. 4). We analyzed more than 10 independent hairy root lines for the TAG accumulation as a function of *GmDGAT1A* and *2D* over-expression. The ectopic expression of *GmDGAT1A* and *2D* increased TAG contents in these transgenic hairy roots by about 2 to 4 folds as compared with GFP vector control, indicating that overexpression of *GmDGAT1A* and *2D* triggered a *de novo* TAG biosynthesis (Fig. 4). Analysis of FA compositions in these TAGs suggested that *GmDGAT2D* expressed in soybean hairy roots preferred to synthesize TAGs with linoleic acid (18:2) acyl chains; meanwhile slight decreases in 18:0- and 18:1-TAGs were observed. The 16:0 content decrearsed averagely by 11%, 18:0 decreased averagely by 21%, 18:1 decreased by 28%, but 18:2 averagely increased by 55% (Fig. 4d). *GmDGAT1A*-hairy roots preferred to synthesize TAGs with linolenic acid (18:3) acyl chains and to less extent, 18:2 acyl chains, at the expense of 18:1 (Fig. 4f,g). The portion of 18:2 in *GmDGAT1A*-transgenic hairy roots increased by 13–40%, whereas 18:3 increased by 46–120%, and meanwhile, 18:1 decreased averagely by about 47% (Fig. 4g).

### *GmDGAT* expression in *Arabidopsis thaliana* alters seed oil production and TAG composition

**Figure 5:**
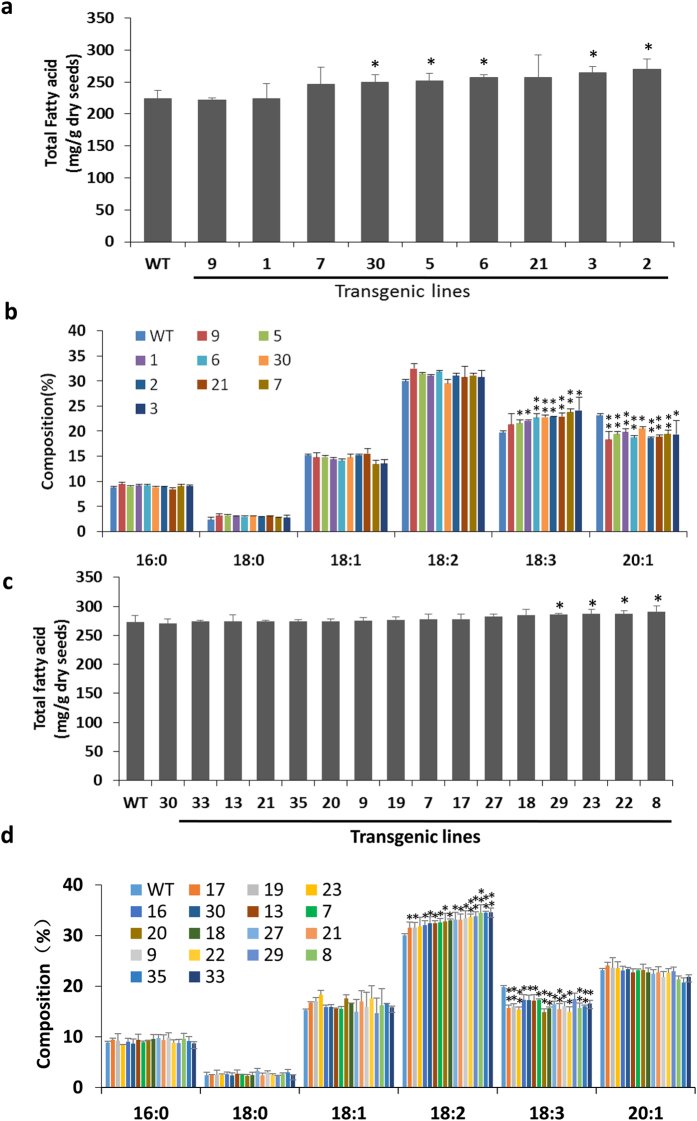
Functional expression of *GmDGAT-1A and -2D* in *Arabidopsis thaliana* seeds. (**a**) Total TAGs in *GmDGAT1A*-expressing Arabidopsis seeds. (**b**) Fatty acid composition of TAGs from Arabidopsis seeds expressing *GmDGAT1A.* (**c**) Total TAGs in *GmDGAT2D*-expressing Arabidopsis seeds. (**d**) Fatty acid composition of TAGs from Arabidopsis seeds expressing *GmDGAT2D.* All data are expressed as mean ± SD from at least three biological duplicates. *P < 0.05 and **P < 0.01 by Student’s *t* test for significant difference. T2 transgenic Arabidopsis seeds were used for analysis.

In order to verify the function of GmDGAT1A and 2D in seed oil production, *GmDGAT1A* and *2D* was also expressed in Arabidopsis Col-0. Analysis of seeds from T3 plants of more than 10 independent lines expressing *GmDGAT1A* and *2D* as confirmed by qRT-PCR (Supplementary Fig. S3). From more than 10 independent lines, the markedly increased amounts of 18:2 were detected in *GmDGAT2D*-expressing Arabidopsis seeds relative to the seeds from control plants. The 18:1 content also increased in all lines, but 18:3 drastically reduced in transgenic lines, averagely by 18%. The highest total contents of 18:1 and 18:2 detected in seeds of transgenic Arabidopsis lines were approximately 20% more than these in seeds of control plants (Fig. 5).

For *GmDGAT1A*, the markedly increased amounts of 18:3 (averagely by 15%) were detected in *GmDGAT1A*-expressing Arabidopsis wild-type Col-0 seeds relative to the seeds from control plants (Fig. 5b). 18:2 content also increased by 4%, whereas 20:1 decreased markedly by 17%. The total TAG contents in these *GmDGAT1A*- or *GmDGAT2D*-transgenic lines are also enhanced to different degrees as compared with the wild-type Arabidopsis seeds (Fig. 5ac).

**Figure 6:**
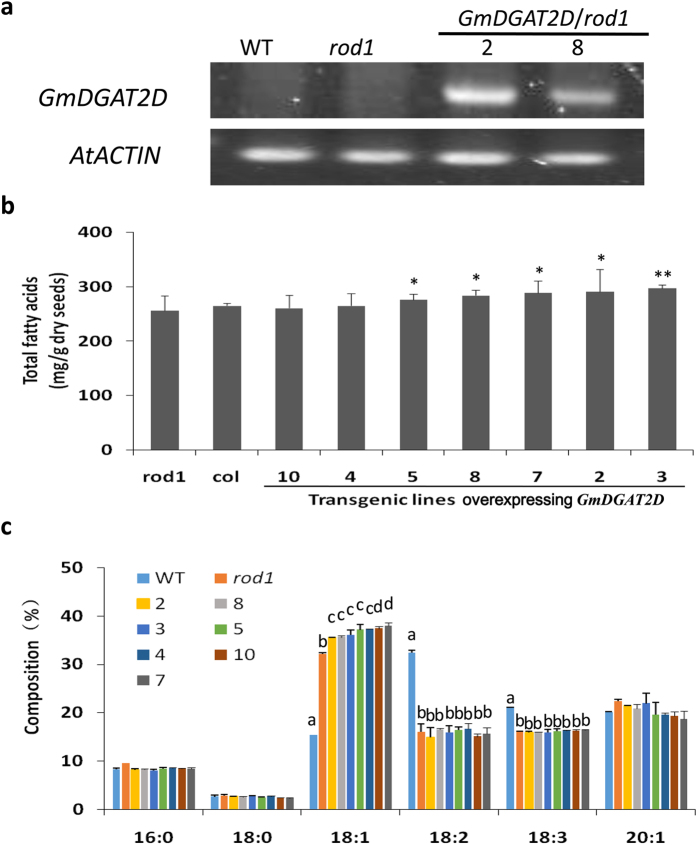
Effects of *GmDGAT2D* overexpression on TAG production in *rod1* mutant seeds. (**a**) Expression of GmDGAT2D in *rod1* mutants, as confirmed by semi-quantitative PCR. (**b**) Total TAGs in *GmDGAT2D*-expressing Arabidopsis *rod1* mutant seeds. *P < 0.05 by Student’s *t* test (n > 3) for the significant difference. (**c**) Fatty acid composition of TAGs from Arabidopsis wild-type (Col-0) seeds, *rod1*, and *rod1* expressing *GmDGAT2D*. Different letters in a group indicate sample with significant differences (p < 0.05) from each other. Data are expressed as mean ± SD from at least three biological duplicates. T3 transgenic Arabidopsis seeds were used for analysis.

To gain more insights into the properties of GmDGAT2D, *GmDGAT2D* was overexpressed in Arabidopsis mutant *rod1 (reduced oleate desaturation 1*) plants. *ROD1* encodes a phosphatidylcholine:diacylglycerol cholinephosphotransferase, which catalyzes a major reaction transferring 18:1 into PC for desaturation and also for reversely transferring 18:2 and 18:3 into DAG for the TAG synthesis pathway^37^. Therefore, the seeds of *rod1* mutant have an averagely 60% reduction in 18:2 plus 18:3 but a similar portion of increase in 18:1TAG^37^ (Fig. 6c). When *GmDGAT2D* was over-expressed in *rod1* mutant, although total oil production averagely increased by only 5.7% in these transgenic seeds of T3 generation, the 18:1 increased by about 14% averagely than these in *rod1* seeds, or increased by about 2.4-fold compared with the wild-type control. Meanwhile, the contents of 16:0 were reduced by 14%, compared with these in *rod1* mutant seeds (Fig. 6b,c).

### Expression of *GmDGAT1A* and *2D* in response to environmental and hormonal stresses

**Figure 7:**
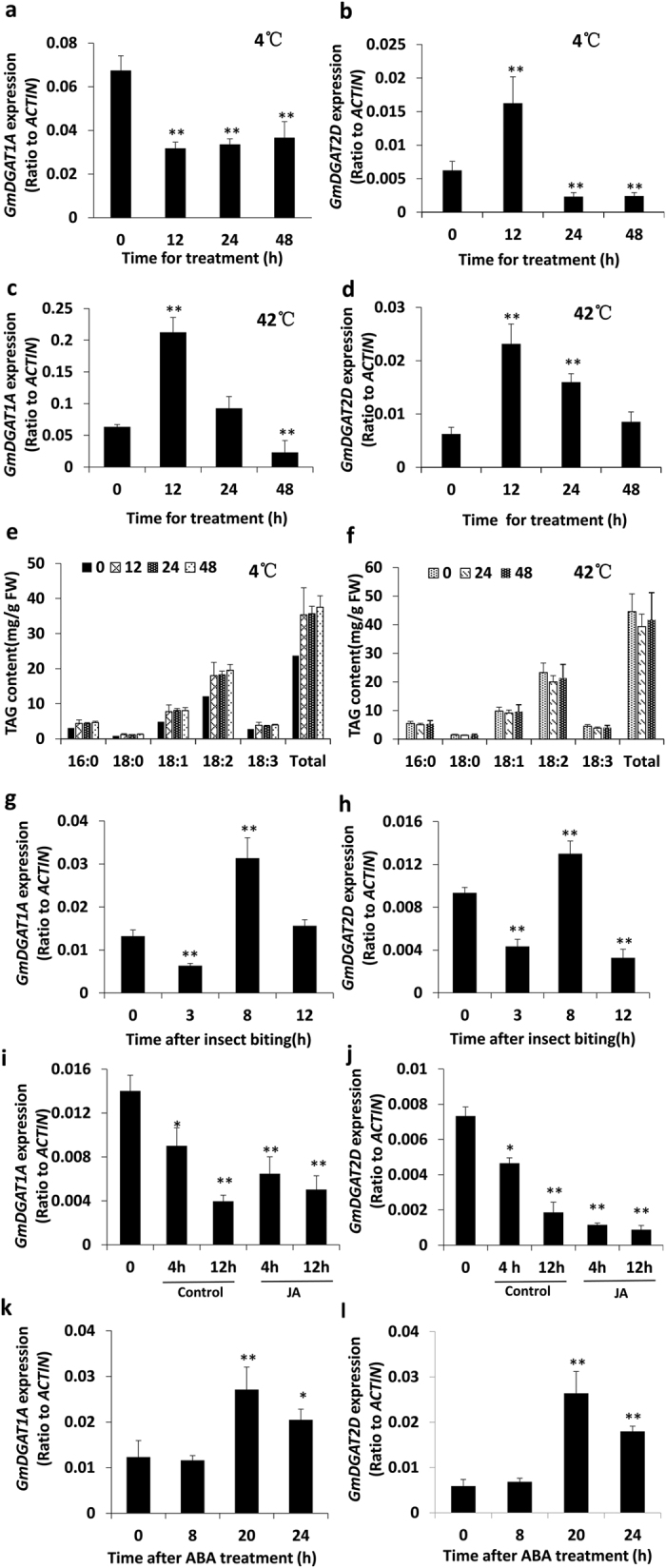
Expression of *GmDGATs* in response to hormonal and environmental stresses. Soybean seedlings with 9 trifoliate (4 weeks old) were subject to insect biting, spraying with hormones (100 μM ABA), or their detached leaves were floating on 50 μM MeJA solution and water (control). Eight-week old soybean plant bearing pods were moved into a cold (4 °C) or heat (42 °C) stress in incubator for indicated time. Leaf samples or pods were harvested at the indicated time. qRT-PCR analysis of *GmDGAT1A* (**a,c,e,g,i,k**) and *GmDGAT2D* (**b,d,f,h,j,l**) expression under abiotic and biotic stresses. Soybean developing seeds treated under 4 °C (**a,b**) or 42 °C (**c,d**) stresses. Total TAG contents and fatty acid composition of soybean seeds exposured to 4 °C (**e**) or 42 °C (**f**) for the indicated time. Soybean seedlings were subject to insect biting (**g,h**), JA treatment (**i,j**), ABA treatment (**k,l**). Data are expressed as mean ± SD (n > 3). *P < 0.05 and **P < 0.01 by Student’s *t* test for significant difference.

*GmDGAT2D* was up-regulated by abiotic stresses, such as heat, and cold stress and biotic stresses (Fig. 7, Supplementary Figs S4 and 5). Upon the cold treatment (4 °C), *GmDGAT2D* transcript levels in developing seeds initially increased at 12 h after cold treatment, and then droped markedly, whereas it was not the case for *GmDGAT1A,* which was down-regulated upon cold treatment (Fig. 7a,b). Also, under heat stress at 42 °C, *GmDGAT1A* and *2D* expression in seed also showed different patterns: *GmDGAT2D* transcripts increased firstly and then fell back to the basal level, whereas *GmDGAT1A* transcript level increased and then droped to a level lower than the basal at 48 h post treatment (Fig. 7c,d). We then examined the TAG biosynthesis in seeds under cold and heat treatments. Coincident with the increase in *GmDGAT2D* transcripts, TAG increases were observed after soybean seeds were subject to cold treatment (Fig. 7e). Meanwhile, contents of 18:1 and 18:2 FAs increased in TAGs. However, under heat stress, only 18:1 consistently increased while 18:3 decreased, and the contents of total TAG remained unchanged (Fig. 7f).

Both *GmDGAT1A* and *2D* transcripts were down-regulated by insect biting and MeJA treatment. The insect biting on the soybean leaves for 4 h and 6 h resulted in a drastic decrease in *GmDGAT2D* transcripts, *GmDGAT1A* transcripts showed fewer decrease (Fig. 7g,h). The detached leaves floating on MeJA solution for 4 h, 8 h, and 12 h, also have consistently down-regulated *GmDGAT2D* and *1A* transcript levels, with *GmDGAT2D* transcripts decreased to a much greater extent than *GmDGAT1A* transcripts did. While leaves floating on water showed less reduction in two *GmDGAT* transcripts (Fig. 7i,j). ABA treatment of seedlings also altered both *GmDGAT* transcripts, with marginal changes at 8 h, and then increased at 20 h after treatment, followed by decreasing at 24 h post treatment (Fig. 7k,l). Again, *GmDGAT2D* transcripts increased more significantly than *GmDGAT1A* did.

## Discussion

We characterized and compared the functions of two soybean DGATs, GmDGA1A and GmDGAT2D, in TAG biosynthesis by using yeast mutant complementation, ectopic expression in soybean hairy roots, and overexpression in Arabidopsis seeds. It is concluded that GmDGAT2D is a highly active type 2 DGAT involved in regular soybean TAG biosynthesis. The both ER-localized GmDGAT2D and 1A catalyzes regular TAG biosynthesis with distinguishable preferences to utilize 18:1 and 18:2 acyl CoA (GmDGAT2D) or 18:3 acyl CoA (GmDGAT1A) as acyl donors. Since most reported plant DGAT2s are involved in biosynthesis of unusual TAG with hydroxy FAs, this soybean GmDGAT2D is thus different from these plant DGAT2s, but more close to animal, fungal or algae DGAT2s, which have potential for enhancing oil production and improve oil compositions in crops^7^,^28^,^38^.

### *GmDGAT1A* and *2D* are ER-localized functionally active short polypeptides

Both GmDGAT1A and 2D can complement TAG-deficiency mutant H1246 phenotype, suggesting they are functional DGATs. GmDGAT2D had much stronger activity in yeast TAG biosynthesis than GmDGAT1A did. Both GmDGAT1A and GmDGAT2D do not possess the known ER retrieval motifs –K(X) KXX–COOH, nor the motif YKSKW of the C-terminus^20^. GmDGAT2D is a shorter polypeptide of 329 amino acids, as compared with GmDGAT1A of 500 amino acids. GmDGAT2D only has two TM domains whereas GmDGAT1A has eight TMs. We showed that GmDGAT1A- and GmDGAT2D-GFP similarly locate to the ER when expressed in tobacco leaf epidermal cells (Fig. 2). Unlike fungal DGAT2s, such as NcDGAT2 and TaDGAT2, that usually have longer and unique N-terminal extension (around 200 aa), GmDGAT2D does not have it^20^,^38^. The histidines in active sites and N-terminus being essential for formation of DGAT dimmers and tetraimers, and a phenylalanine essential for a maize DGAT activity are present in GmDGAT1A and 2D^20^,^39^. From results in yeast, soybean, and Arabidopsis expression of *GmDGAT2D*, we conclude that GmDGAT2D is different from other known plant DGAT2 for unusual oil synthesis: GmDGAT2D is more similar to DGATs from fungi, animal, and some plants such as Arabidopsis and flax^10^,^11^,^24^,^26^, for regular TAG biosynthesis^28^,^29^. Also, GmDGAT2D appears to have subdomain localizations in the ER similar to these of GmDGAT1A, which may suggest similar functions of Type 1 and 2 DGATs in TAG biosynthesis. This is different from a previous report on Tung tree DGAT1 and DGAT2^10^.

### GmDGAT1A and 2D have different substrate preferences for regular TAG biosynthesis

GmDGAT1A and 2D restored TAG biosynthesis in *S. cerevisiae* strain H1246, they are functional DGAT enzymes. We also found that expression of *GmDGAT2D* in both soybean hairy roots and Arabidopsis wild-type plants increased TAG contents and changed TAG compositions. An increased 18:2 and 18:3 acyl chains in TAGs was observed in *GmDGAT2D* and *GmDGAT1A* transgenic hairy roots, respectively. Overexpression of *GmDGAT1A* and *2D* in Arabidopsis seeds results in substantial changes in TAG contents. A significant increase in 18:2 and slight increase in 18:1 acyl chains in TAGs, accompanied by a pronounced decrease in 18:3, were observed with *GmDGAT2D* transgenic lines. In contrast, 18:3 was significantly increased, accompanied with decrease in 20:1 or 18:1 in *GmDGAT1A*-transgenic Arabidopsis seeds or soybean hairy roots, respectively. These data suggest that GmDGAT1A has a strong preference to 18:3 acyl-CoA and GmDGAT2D prefers to use 18:2 and then 18:1 acyl-CoAs. However, when *GmDGAT2D* was over-expressed in *rod1* mutant, whose seeds accumulate much more 18:1 than 18:2, *GmDGAT2D/rod1* seeds produced even more 18:1 than *rod1* mutant seeds, but 18:2 contents were unchanged. The data suggest that substrate preference for DGATs, at least for GmDGAT2D, is dependent of availability of preferrd substrates. GmDGAT2D is more similar to DGAT2s from fungi and animals in regular TAG synthesis in plants^20^,^38^,^40^, but unlike other DGAT2s in plants that are involved in synthesis of unusual TAGs containing hydroxy FAs^7^,^24^,^26^.

The substrate preferences of DGATs can significantly edit the acyl chains in TAGs, which may mark plant species with specific features in storage TAGs. The analysis of the neutral lipids from the *GmDGAT1A and 2D*-transformed yeast strain revealed that GmDGAT1A and 2D-catalized TAG production with more 16:1 and 18:1 unsaturated FA chains, as compared with native TAGs with 16:0 and 18:0 saturated and 18:1 unsaturated FAs in yeast. When expressed in Arabidopsis seeds, *GmDGAT2D* render the transgenic Arabidopsis to accumulate significantly more 18:2 and slightly increased 18:1 TAGs in seeds, in expense of a reduced 18:3 TAGs. However, *GmDGAT1A* renders transgenic Arabidopsis to accumulate significantly more 18:3 in seeds, but a reduced 20:1 TAGs. Thus two DGATs showed very different substrate preference and therefore transgenic concequences. Results from transgenic soybean hairy roots also provided unambiguous evidence that GmDGAT2D preferentially utilizes 18:2 acyl-CoA as acyl donors for TAG biosynthesis, whileas GmDGAT1A prefers to utilize 18:3 acyl-CoA as acyl donors for TAG biosynthesis, This substrate preference may not be unexpected, given that no 18:2 available in yeast cells, but 18:2 accounts for around 25–30%, and 18:3 (~20%), of the total FAs in soybean and Arabidopsis seeds, whereas 18:1 are relatively minor unsaturated FAs (~17%)^2^,^20^. However, in soybean hairy roots, 16:0 and 18:2 FAs account for about 30% and 25%, respectively, of total FAs, whereas 18:0, 18:1, and 18:3 each accounts for only around 15–18%. Therefore, GmDGAT2D selects 18:2 for TAG synthesis in soybean hairy roots, but selects 18:1 in *rod1* mutant seeds (accounting for more than 40% of total FAs). GmDGAT1A prefers to use 18:3 for TAG biosynthesis, although 18:3 FAs are small portion. Therefore, both GmDGATs prefer to use unsaturated FAs as substrates in yeast and plant cells. The different substrate preferences of GmDGAT2D and GmDGAT1A may confer the diverse FA profiles in soybean oils. Meanwhile, their differential expression patterns also indicate that they may have other additional functions in non-seed tissues upon stresses.

### Role of GmDGAT2D in plant response to stresses and GmDGAT1A in seed TAG biosynthesis

It is well-known that oil production in oilseed is regulated by ABA signaling, which regulates seed maturation at least through abscisic acid insensitive 4 (ABI4) and downstream transcriptional machineries^5^,^41^. *GmDGAT2D* transcripts are also found in non-seed tissues, such as flower, root and leaf, where it may play a role in glycerolipid biosynthesis and physiological response to adverse environments, although functions of TAG and DGATs in these tissues remain ambiguous^42^,^43^. In flower, coat materials in the pollen grain surface include TAGs, other lipids, and flavonoids synthesized by the tapetum cells for keeping pollen viability under stress conditions^6^,^19^. Anther filament elongation plays an essential role in plant pollination. Failure in anther filament growth, one of the reasons for plant male sterility, may be attributable to JA signaling pathway^44^. JA is required for anther filament development, and blocking JA synthesis or signaling (e.g. *coi1* mutant) confers male sterile as a result of improper anther filament elongation^44^,^45^. COI1 is specifically expressed in the anther filament where *GmDGAT2D* is also highly expressed, indicating a possible connection between JA signaling and GmDGAT2D and TAG biosynthesis. *GmDGAT2D* transcript levels coincidently decreased followed by insect biting, wounding, and MeJA treatments. The insect biting caused-wounding effect usually induces an endogenous JA increase in plant tissues, as one of mechanisms to initiate plant responses^6^,^44^,^45^,^46^. This may explain that both insect biting and MeJA treatment similarly repressed *GmDGAT2D* expression.

Since plants normally accumulate more polyunsaturated FAs for glycerolipid biosynthesis in response to cold stress^47^, and GmDGAT1A and 2D prefer to utilize 18:3- and 18:2-CoA for TAG biosynthesis, it is plausible that *GmDGAT2D* was up regulated in cold and heat stresses and contributed to adaptation of plants to these stresses by modifying the polyunsaturated TAG synthesis. *GmDGAT2D* promoter contains several ABA and JA responsive *cis*-elements, such as DPBFCOREDCDC3, MYCCONSENSUSAT, MYCATRD22, and PYRIMIDINEBOX that are responsible to cold stress and ABA signaling (Supplementary Table S2). However, the promoter of *GmDGAT1A* has fewer ABA and JA responsive cis-elements, consistently; *GmDGAT1A* transcripts were less changed upon JA treatment,

TAG usually represents <1% of leaf glycerolipids but can accumulate more under stresses, such as heat and cold treatments, or other conditions such as expression of structural or regulatory genes^6^,^43^,^47^. Recently, it was found that DAG and TAG are also identified from phloem exudates under normal and stressed conditions, suggesting that they might act as long distance signals^46^. However, what is TAG biological fuctions under these conditions and which DGAT is responsible for spatial and temporal TAG biosynthesis under normal or stressed conditions is not understood^43^,^47^. Given stress-responsive *GmDGAT2D* or *GmDGAT1A* with such dramaticly changed expression patterns under various conditions in this study, and recent reports showing lipid metabolism is involved in multiple biotic and abiotic stress responses in plants^47^, DGATs and TAG metabolism may have intriguing connections with plant-environment interactions. However, whether *GmDGAT2D* expression in anther filament is also involved in production of these vascular long-distance traveling TAGs remain to be determined.

The similarly steady increases of *GmDGAT1A* and *2D* expression levels at the stages of active oil accumulation, followed by the decreases when TAG reaches the plateau, suggest that GmDGAT1A and 2D play essential roles in soybean seed oil production. This is in contrast to oleogenic seed crops that contain unusual FAs, where DGAT2 plays a more central role than DGAT1 in unusual oil production^10^,^24^,^26^. Since soybean oil does not contain unusual FAs, as the major contributors of TAG production, GmDGAT1A and 2D, together with other DGATs, function redundantly in TAG biosynthesis in both seeds, flowers, leaves, and other tissues under specific conditions^43^.

Plant employs different strategies to respond to environmental stresses, such as cold and heat, during the seed development and seed filling, when metabolism of polyunsaturated FAs is believed to play an essential role in adaptation to these stresses^4^,^47^. The differential responses of various DGATs to heat and cold stresses suggest that they may function in fine tune to change lipid metabolic pathways correspondingly to ensure the proper TAG biosynthesis for adapting them to temperature changes^4^,^6^,^47^. Given the increasing concerns on effects of unsual global climate changes, such as frequently high temperature and cold stresses, on soybean production, further investigation on regulation of DGATs and TAG metabolism may provide insights into how plant glycerolipid production is affected by environmental stresses^6^,^47^.

### Potential application of two GmDGATs for metabolic engineering of soybean oil composition

The considerable attention has been paid to DGATs as biotechnological targets for improvement of the TAG production in oilseed crops such as soybean, maize, and rapeseed to meet the growing demand for vegetable oils or biofuels^13^,^27^,^38^. Seed-specific overexpression of Arabidopsis DGAT1 increased oil content by up to 15 and 46% in seeds of transgenic *Brassica napus* and Arabidopsis, respectively^13^,^48^. The increased oil contents in maize and soybean seeds were also archived by manipulating the expression of the fungus *Umbelopsis ramanniana* UrDGAT2 or algae DGAT2^7^,^27^,^38^. Most DGAT2s from plants such as *R. communis* and *Vernonia galamensis* are capable of using hydroxyl or epoxyl fatty acyl-CoAs (e.g. ricinoleic acid, vernolic acids) into TAGs to produce unusual TAGs for industrial purposes^24^,^26^, while we show that GmDGAT2D is different from them.

Because higher levels of 18:3 in soybean seeds is a major cause for oxidation instability of vegetable oils, and *trans*-fats generated primarily in hydrogenation of vegetable oils during food processing have negative impact on human health, enriching 18:1 and 18:2 and meanwhile minimizing 18:3 in vegetable oils has become one of targets for crop biotechnology^2^. Our study here revealed two soybean DGATs, GmDGAT2D are responsible for 18:1 and 18:2 production and GmDGAT1A for 18:3 in soybean seeds. The demonstration provides possible strategies for either increasing 18:1-TAG and repressing 18:3-TAG production in a more specific way than the reported technology for high-oleic acid soybean variety^37^,^38^,^40^.

The study sheds light on our understanding of TAG biosynthesis and metabolic engineering of soybean oil with appropriate DGATs. Using a stronger seed-specific promoter may further improve the performance of GmDGATs for creating soybean oils with desired composition. Moreover, our study suggests roles of TAG biosynthesis and DGATs in other non-seed tissues during plant responses to hormones and various stresses, which may open a door for exploring and understanding more fundamental lipid biology in crops.

## Methods

### Plant growth conditions and treatments

Soybean (*Glycine max* L.) seeds were germinated in three-gallon pots containing soils. The seedlings were grown in growth chamber (26/20 °C day/night temperature, photoperiod of 14/10 h, 800 μmol m^−2^ s^−1^ light intensity and 60% humidity). Roots, leaves, stems, flowers, seeds, and drupes at different developmental stages were harvested from “Tianlong No.1”, a soybean (*Glycine max* L.) cultivar, grown in a growth chamber under the same conditions as above or a natural environment at the fields of Huazhong Agricultural University at Wuhan, China. For methyl jasmonate (MeJA) treatment, soybean leaves was detached and floated on the water containing or not containing 100 μM MeJA. For insect biting experiments, the chewing insect measuring worms (*Geometridae)* were put on four-week old soybean plants with 3- trifoliate leaves for insect biting. For cold and heat treatments, eight-week old soybean plants bearing pods were moved into 4 °C refrigerator with regulator lights or into oven set at 42 °C for treatments. Leaves or seeds (at stage 4) were collected at different time and immediately frozen in liquid nitrogen and stored at −80 °C after treatments for RNA extraction and TAG analysis.

### Vector construction

The open reading frames (ORFs) of *GmDGAT1A* (Glyma13G106100) and *GmDGAT2D* (Glyma.01G156000) were amplified with the cDNA made from soybean developing seeds using pairs of primers for *GmDGAT*s (Supplementary Table S1). Total RNA was extracted from *G. max* developing seeds, and 10 μg of total RNA was used to synthesize first-strand cDNA using the first-strand synthesis system (Invitrogen). The cDNA was synthesized as a template for *GmDGAT2D* cDNA amplification. After *GmDGAT1A* and *GmDGAT2D* ORFs was cloned into T-easy vector and sequenced, the cDNAs in pDONOR221, pDONOR221-*GmDGAT1A* or -*GmDGAT2D,* was recombined into different destination vectors such as pYESTDEST52 and pB2GW7 by using LR recombinase (Invitrogen), and were sequenced. For *GmDGAT2D* promoter analysis, a 1.5 K bp promoter region was amplified with primers proF and R (Supplementary Table S1). The PCR products were cloned into T-easy vector for sequencing; then subcloned into pDONOR221 and subsequently pHGWFS7 by using BP and LR recombinases (Invitrogen).

### Soybean hairy root transformation and analysis of TAG in hairy roots

*pB2GW7-GmDGAT1A or -GmDGAT2D* was transformed by electroporation into *Agrobacterium rhizogene*s strain K599, which was used to transform soybean cotyledons. Seeds of soybean cultivar “Tianlong” was surface sterilized and germinated in sterilized filter papers in petridishs. The green cotyledons from about 7 days-old germinating soybean seeds were wounded on the surfaces, followed by the infections with *Agrobacterium rhizogene*s K599 bacteria harboring *pB2GW7-GmDGAT1A, -GmDGAT2D,* or *-GFP* as a control. Generated hairy roots were selected on MS medium containing antibiotics for controlling Agrobacterium and 7 mg/l phosphinothricin (ppt) for transformed hairy roots. The transformed hairy roots expressing *pB2GW7-GmDGAT1A or -GmDGAT2D* were confirmed with PCR. Then roots were used for extraction of total lipids, from which TAG was separated on TLC. TAG spots were scraped off the plate and extracted for measurement by using GC.

### Expression of GmDGATs in Arabidopsis

The binary vector containing the cassette for *35S::GmDGAT1A* or *35S::GmDGAT2D* was transformed into *Agrobacterium tumefaciens* GV3101 by electroporation. Transgenic Arabidopsis Col-0 plants were transformed by using floral dip method. BASTA was used for selection of transformant seedlings which were also PCR confirmed using 18s univ F and 18s univ R, as well as together with gene specific 3′ primers (Supplementary Table S1). Expression of the transgene in developing seeds was confirmed by RT-PCR. The dry seeds of T3 Arabidopsis transformants were analyzed for oil contents and FA composition.

### Yeast strains and transformation

For the functional expression of GmDGAT1A or 2D in yeast, the quadruple mutant Saccharomyces cerevisiae strain H1246 (W303; MATα are1-Δ::HIS3 are2-Δ::LEU2 dga1-Δ::KanMX4 lro1-Δ::TRP1 ADE2 ura3)^34^ and the wild-type S. cerevisiae strain YPH499a (ura3-52, lys2-801, ade2-101, trp1-63, his3-200, leu2-1) were used as heterologous hosts. The plasmid DNA pYESDEST52-GmDGAT1A or 2D was transformed into stationary H1246 cells using the PEG/lithium acetate method^49^. Yeast cells harboring the empty pYESDEST52 vector were used as negative control. Transformants were selected on YNB lacking uracil and functional expression and induction of GmDGAT1A or GmDGAT2D was performed as described previously^50^.

### Yeast expression and Nile red assay

Intracellular lipid bodies were visualized by fluorescent microscopy using Nile Red (9-diethylamino-5H-benzo α-phenoxazine-5-one, 5 ul Nile Red in 0.8 mg/mL acetone stock) staining method^51^,^52^. Fluorescence microscopic images of the stained cells were recorded using excitation wavelength at 485 nm and emission wavelength at 538 nm under Nikon Eclipse 80I microscope.

Nile red assay and optical density determination were performed in 96-well polystyrene flat-bottom microplates (UNIPLATE, Whatman, UK), according to previously described^52^. The Nile red fluorescence assay was performed at excitation and emission filters of 485 and 538 nm, respectively. The assays were performed with ten technical replicates. The measurement of the fluorescence intensity of various yeast cultures were done after adjusting these yeast resuspension cells to OD_600 nm_ to 1 to make sure that all comparisons are in the same cell density.

### Quantification of TAG and analysis of FA composition

Total lipid extraction and TAG content and composition determination were done according to previously described methods with slight modifications^35^,^52^,^53^,^54^. Briefly, total lipids in yeast pellets (~0.014 g dry yeast) or soybean hairy roots (~0.2 g fresh tissues) were extracted with 4 ml of 4M HCl in glass tubes tightly with Teflon-lined caps at room temperature for 30 min, then in a 100 °C water bath for 10 minute. After cooling tubles were centrifuged, yeast pellets or hairy root powder were extracted with 4 ml of hexane: isopropanol (3:2, v/v). The upper hexane layer of the extractions was removed into a new glass tube and evaporated under a slow stream of N_2._ The residues were dissolved in 50 μl hexane for TLC analysis.

The TAG from non-seed tissues including yeast and soybean hairy roots was resolved by TLC on a silica plate (SIL GF254, 0.25 mm). The plate was developed with hexane/diethyl ether/acetic acid (80:20:1, v/v/v), essentially according to the method as previously described^35^. Fatty acid methyl esters (FAMEs) were prepared by heating the dry TAG materials at 85 °C for 30 min in 1 M HCl in dry methanol, as described by Wang and Benning^52^. FAMEs then were dried under nitrogen gas and resuspended in 200 μl of hexane for GC analysis.

The TAG content and composition from soybean and Arabidopsis seeds were measured according to methods described by Christie^54^. Briefly, ~10 mg of seeds were weighed in a 13×100 mm glass screw-cap test tube. To each tube, 1.5 ml of 2.5% sulphuric acid in methanol, 400 μl toluene, and 100 μl of 1 mg/ml triheptadecanoin in toluene (Nu-Chek Prep, Elysian, USA) as an internal standard were added. The tubes were heated at 90 °C for 1 h. FAMEs generated by above transesterification reaction were extracted by addition of 1.8 ml H_2_O and 1 ml heptanes, and the heptane layer was recovered and analyzed with GC^53^.

FA content and composition on TAGs from seeds or purified with TLC from total lipids of yeast cells and hairy roots were analyzed with an Agilent 7890A GC system with flame ionization detector (FID), according to the conditions described previously^53^. Oil content was calculated by FID response of sample components relative to 17:0 methyl ester from the internal standard triheptadecanoin.

### Soybean Illumina expression data analysis

Solexa sequencing libraries for developing seed samples covering two genotypes of soybean varieties at two developmental stages (at around 20 and 48 days after pollination) were generated and analyzed. Data were utilized to quantify the transcriptomic expression of soybean genes (i.e. the number of sequence reads/million reads aligned). Read counts used in expression analyses were based on the subset of uniquely aligned reads that also overlapped the genomic spans of the soybean gene predictions. Read counts for a given sample were normalized by using values for a gene’s uniquely aligned read counts per million reads uniquely aligning within that sample. A total of 51,529 annotated soybean genes (74.5% of the 69,145 putative, annotated soybean genes) were found to be expressed in at least one condition.

### Quantitative RT-PCR (qRT-PCR) analysis of gene expression

Total RNAs from tissues of soybean plants were isolated using TRIzol reagent (Invitrogen, Carlsbad, CA) or RNA isolation kit (Biotech, Beijing) according to the manufacturer’s instructions. For each sample, 10 μg of total RNA were digested with RNase-free DNaseI (Promega, Madison, WI) to remove any genomic DNA contamination. After DNaseI treatment, RNA concentration was determined again using a NanoDrop ND-2000 UV spectrophotometer (Thermo Scientific, USA). First-strand cDNA was synthesized from 2 μg total RNA using the Superscript III first strand synthesis system (Invitrogen). All cDNA samples were diluted 50-fold in sterile water for qRT-PCR reaction. Gene specific primers were listed in Supplementary Table. S1. Soybean *ACTIN* was used as internal control. qRT-PCR reactions were performed in 96-well plates (iQ5 Real Time PCR System; Bio-Rad) for all tissues tested, and data were analyzed according to methods described previously^49^.

### Histochemical analysis

*pGmDGAT2D::GUS* reporter Arabidopsis lines were generated as described previously^49^. T1 seeds were screened with BASTA for selection of transformants. T2 plants were used for GUS activity. GUS staining of transgenic seedlings was conducted as previously described^49^. Samples were photographed using an Olympus szx16 microscope.

### Subcellular localization of GmDGAT1A- and 2D-GFP in planta

Construction of GmDGAT1A- and 2D-GFP fusion was done by using gateway recombination into pK7WGF2 in fusion with N-terminus of GFP. Determination of the subcellular localization of GmDGAT-GFP was performed by using tobacco leaf infiltration following the method described previously^50^. GmDGAT-GFP and ER marker CD3-959: Cherry DNAs were transformed into *Agrobacterium* strain K599, which were used for plant transformation. The acetosyringone-activated bacteria were co-infiltrated into the abaxial epidermal surface of a tobacco leaf with a syringe, and the plant was grown at room temperature for 2 to 3 d before imaging. Imaging of GmDGAT-GFP fusion proteins was performed using a Leica TCS SP2 inverted confocal microscope using a 63×water-immersion objective and Leica Confocal software with an excitation wavelength of 488 nm and emissions collected at 500 nm. CD3-959-mCherry–labeled ER membrane was excited at 543 nm with the argon laser, and emission was detected from 620 to 680 nm. Chloroplasts were visualized at 560 to 610 nm under excitation at 543 nm.

### Bioinformatics analysis

Protein sequences, GmDGAT2D (GenBank accession number: KP752053) and GmDGAT1A (GenBank accession number: KT878751), were obtained from NCBI and phytozome. Transmembrane regions were predicted by the TMHMM Server ver. 2.0 (http://www.cbs.dtu.dk/services/TMHMM/). Amino acid multiple alignments were made with the ClustalW program (http://www.ebi.ac.uk/clustalw/) under default parameters. A phylogenetic tree was constructed using MEGA4. The significance level of the neighbor-joining analysis was examined by bootstrap testing with 1000 repeats.

### Statistic analysis

Most experimental data were obtained from at least three independent experiments and were analyzed using Student’s *t*-test. The significant differences between two tails of data represent 95% confidence limits. Histochemical staining and fluorescence imaging experiments, at least two repeat experiments were done, and representatives of photos or images were shown.

## Additional Information

**How to cite this article**: Chen, B. *et al*. Two types of soybean diacylglycerol acyltransferases are differentially involved in triacylglycerol biosynthesis and response to environmental stresses and hormones. *Sci. Rep.* **6**, 28541; doi: 10.1038/srep28541 (2016).

## Supplementary Material

Supplementary Information
