# Supplementary material for: Two types of soybean diacylglycerol acyltransferases are differentially involved in triacylglycerol biosynthesis and response to environmental stresses and hormones
Source: Sci Rep. 2016 Jun 27;6:28541. doi: 10.1038/srep28541 (PMC4921965; doi:10.1038/srep28541)
Supplement: Supplementary Information [file srep28541-s1.doc]

**Supplementary Data information:**

**Two types of soybean diacylglycerol acyltransferases are differentially involved in triacylglycerol biosynthesis and response to environmental stresses and hormones**

BeiBei Chen^1^, Junjie Wang^1^, Gaoyang Zhang^1^, Jiaqi Liu^2^, Sehrish Manan^1^, Honghong Hu^3^, Jian Zhao^1^*

**Supplemental Figures:**

**Figure S1. Bioinformatics analysis of GmDGATs from soybean and other plants**

(**a**) Unrooted phylogenetic tree of soybean diacylglycerol acyltransferase (DGAT) and other hypothetical or functionally characterized DGATs. The alignment was generated using ClustalW and the unrooted phylogram was constructed by the neighbor-joining method in MEGA6 software.

(**b, c**) Topological analysis of GmDGAT1A and 2D. The transmembrane domains (TMs) underlined were predicted with the TMHMM Server ver. 2.0 (http://www.cbs.dtu.dk/ services/TMHMM/).

(**d. e**) The amino acid alignment of DGAT2D and DGAT1A homologues, respectively. Accession numbers of the sequences are: *Zea mays* ZmDGAT1, ABV91586.1; *Ricinus Communis* RcDGAT1, ACB30543.1; RcDGAT2, ACB30544.1; *Arabidopsis thaliana* AtDGAT1, AEC06882.1; AtDGAT2, AEE78802.1; AtDGAT3, AAP21223.1; *Glycine max* GmDGAT1A, Glyma13g16560; GmDGAT1B, Glyma17g06120; GmDGAT1C, Glyma09g07520; GmDGAT2A, Glyma09g32790; GmDGAT2B, Glyma16g21960; GmDGAT2C, Glyma16g21970; GmDGAT2D, KP752053; GmDGAT2E, Glyma11g09411; GmDGAT3A, Glyma13g17860; GmDGAT3B, Glyma17g04650; *Helianthus annuus* HaDGAT2, ADT91688.1; *Arachis hypoaea* AhDGAT3, ABW34442.1; *Olea europaea* OeDGAT2, ADG22608.1; *Vernicia fordii* VfDGAT2, ABC94474.1; *Oryza sativa japonica* OsDGAT2, NP_001057530.1.


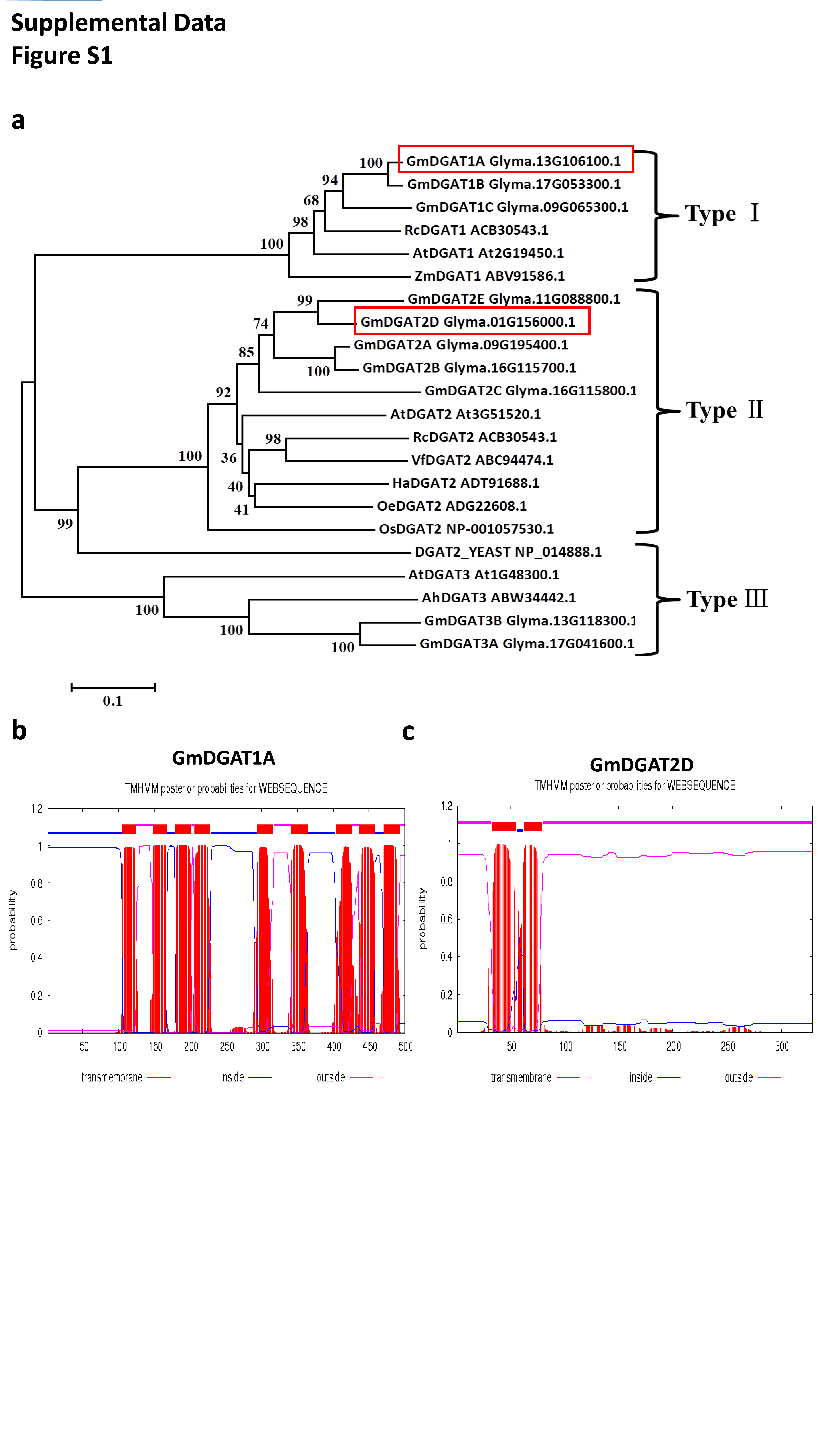


**
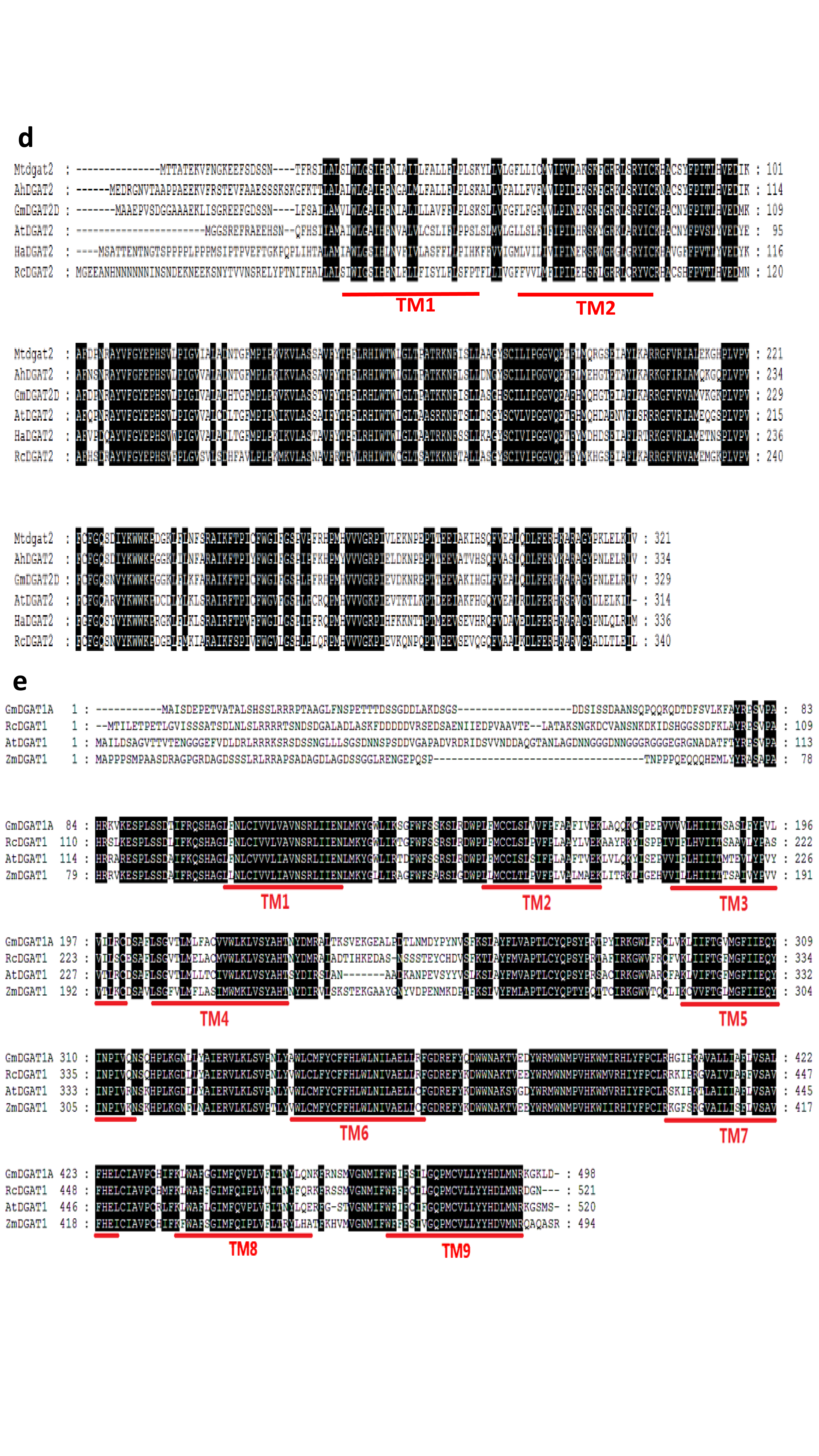
**

**Figure S2. *GmDGAT1A* and *2D* expression patterns in soybean tissues**

**(a).** Expression patterns of *GmDGAT2D* in different tissues of soybean plant. The public data are shown in phytozome (https://phytozome.jgi.doe.gov/). *GmDGAT2D* (*Glyma01g156000 in V10, Glyma01g36011 in V9*).

**(b).** *GmDGAT2D* is also mainly expressed in flower, then in roots, seeds, and nodules, and other tissue. Expression level of *GmDGAT2D* increases steadily during the seed development stage. These data are extracted from soyKB (www. SoyKB.com)

**(c).** Expression patterns of *GmDGAT2D* in root tissues infected with rhizobial bacteria for different times, as compared with other tissues. *GmDGAT2D* is repressed upon rhizobial bacteria infection (IN) as compared with uninfection (UN). Data are from soyKB.

**(d).** Expression patterns of *GmDGAT1A* in different tissues of soybean plant. Data are from phytozome.

**(e).** Comparison of expression patterns of all soybean *DGAT* genes in different tissues. Data are from soyKB.

**(f).** Comparison of expression patterns of soybean *DGAT* genes during seed developmental stages. Data are from soyKB at <http://soykb.org/heatmap2/index.php>).


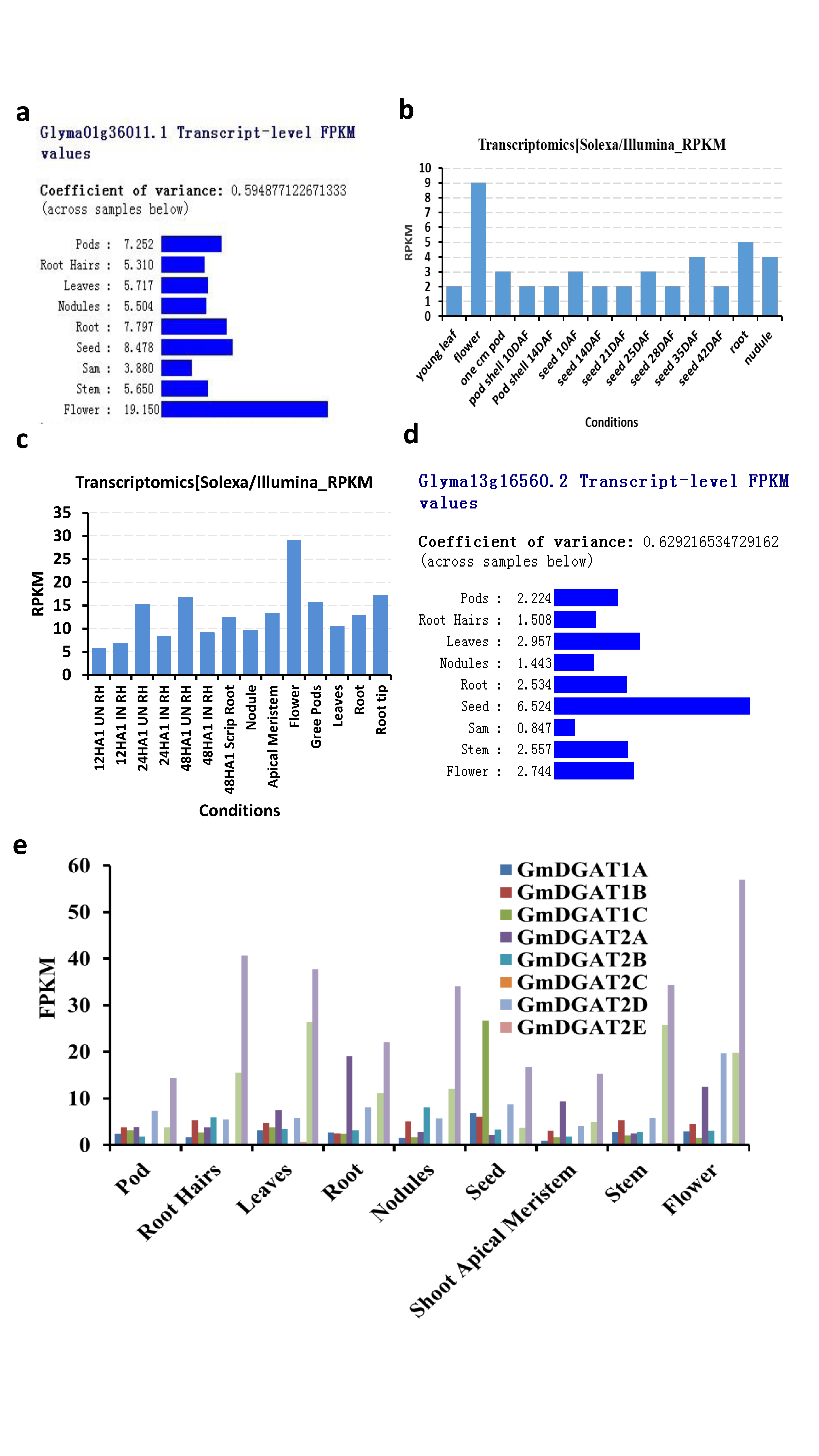


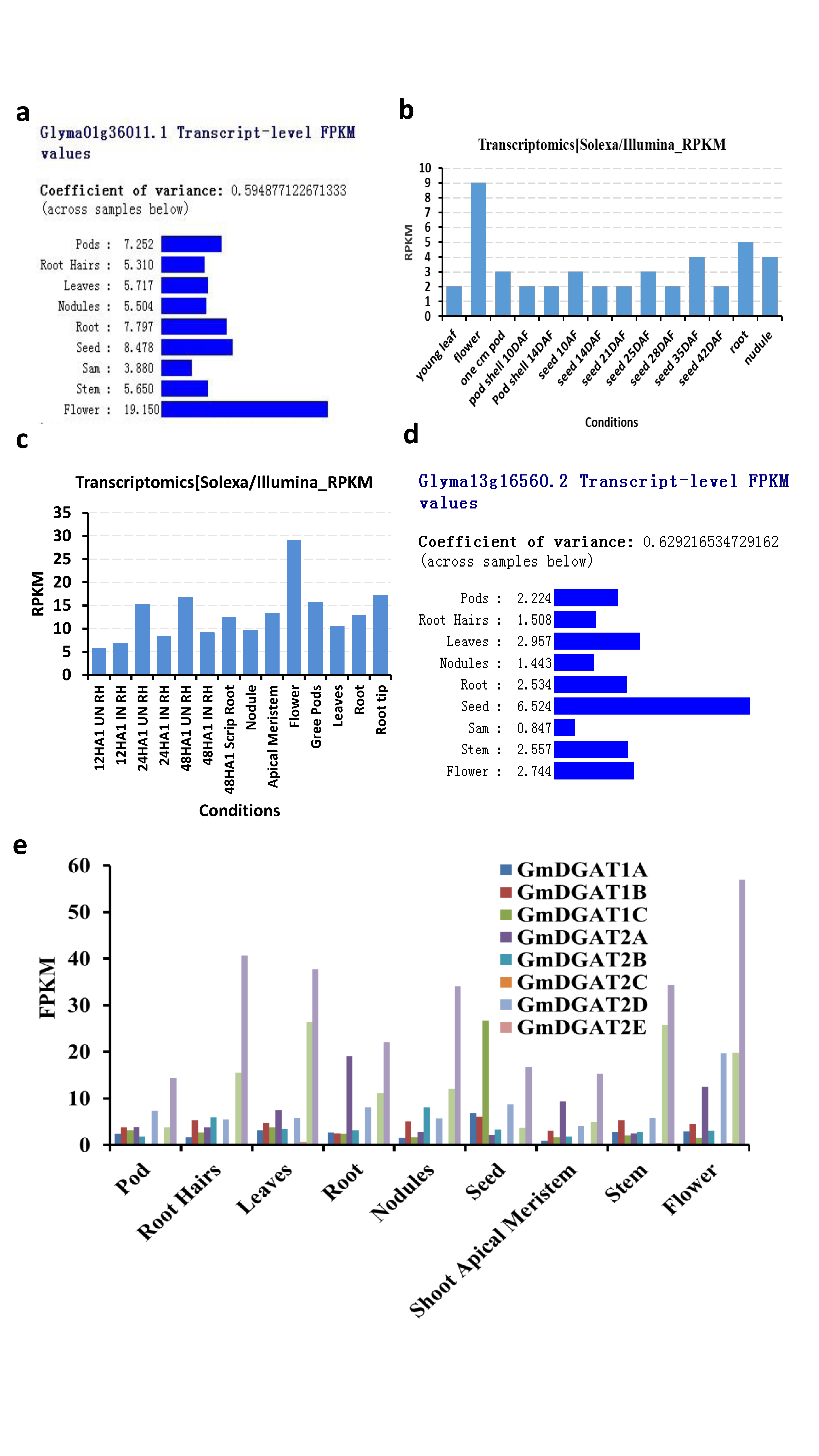


**
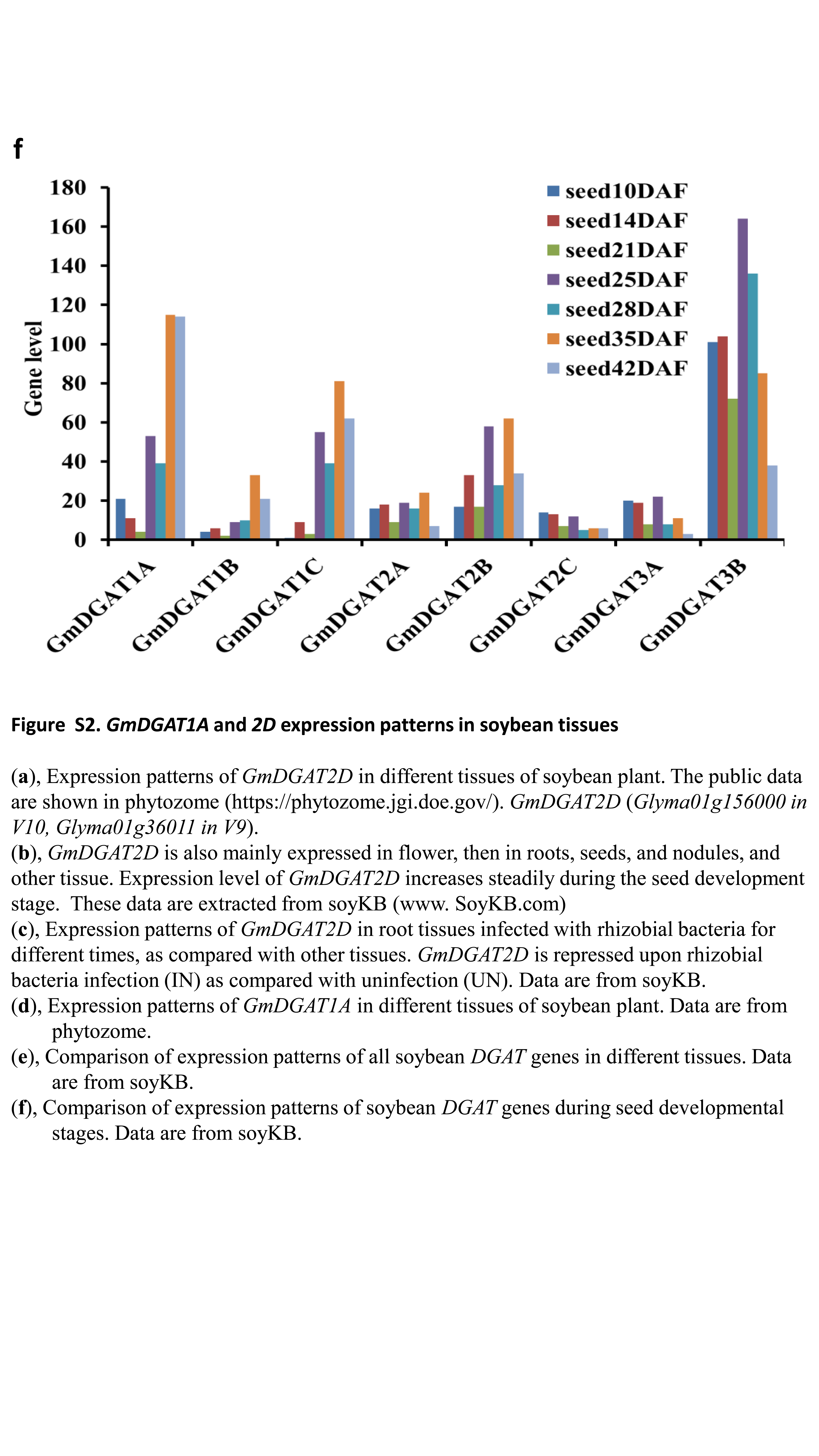
**

**
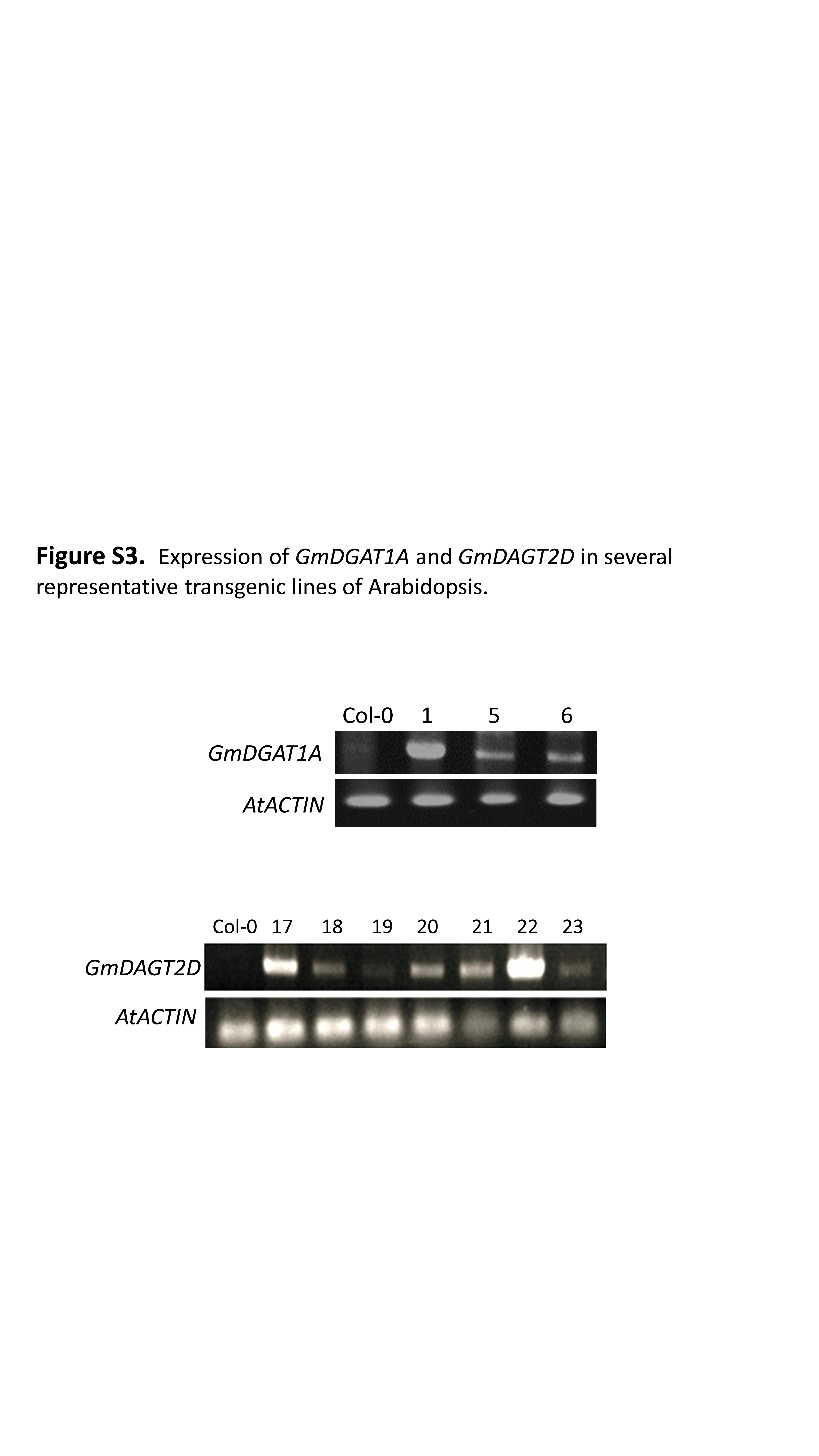
**

**Figure S4.** Expression profiles of *GmDAGT1A* and *2D* in soybean tissues (*GmDGAT2D* was represented by two probe sets, GmaAffx.78871.1.A1_at and GmaAffx.63387.1.S1_at). Data are extracted from public databases on SoyKB.com.

**
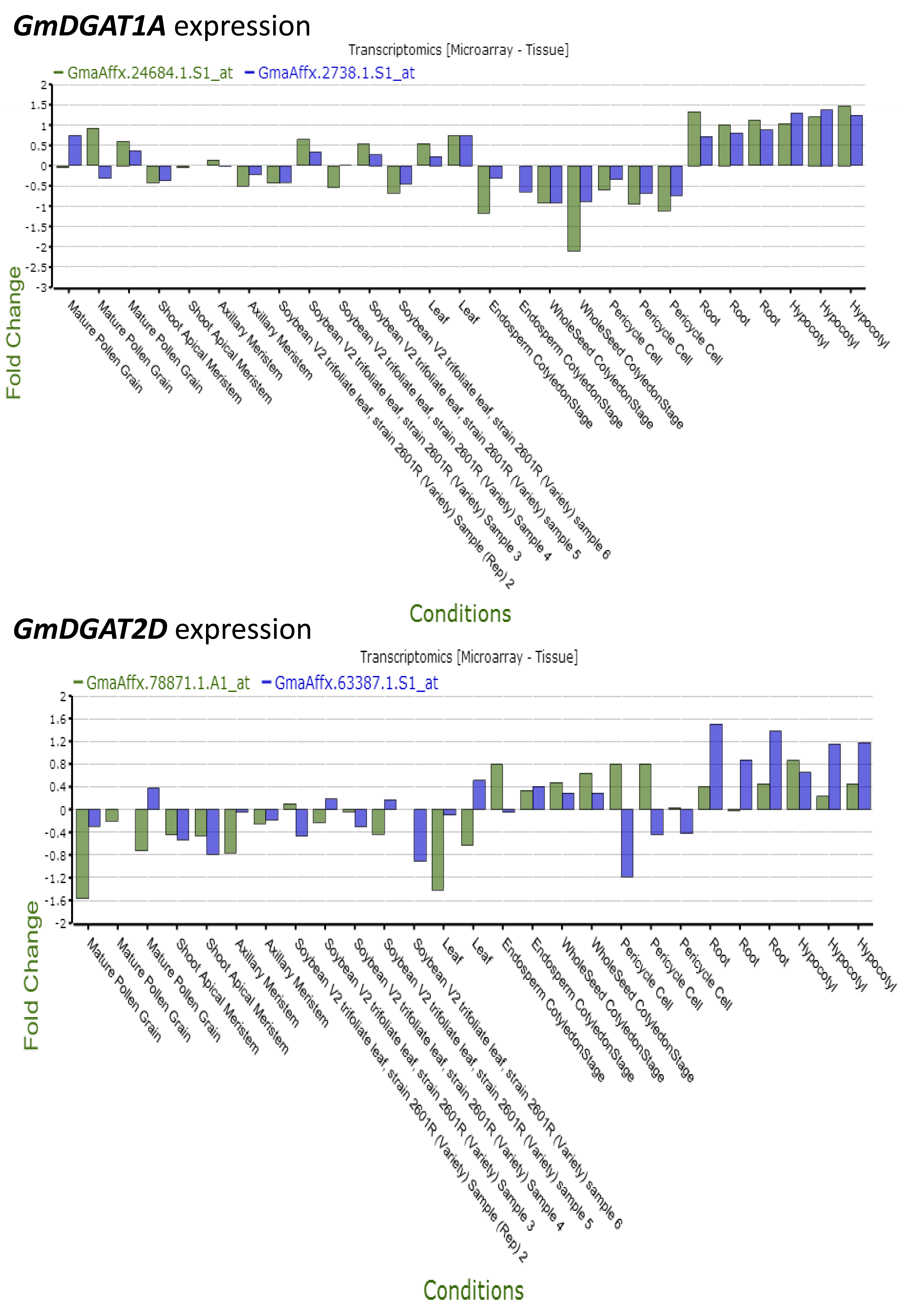
**

**Figure S5.** Expression profiles of *GmDAGT2D* in soybean under various abiotic and biotic stresses (*GmDGAT2D* was represented by two probe sets: GmaAffx.78871.1.A1_at and GmaAffx.63387.1.S1_at ). Data are extracted from public databases on SoyKB.com.


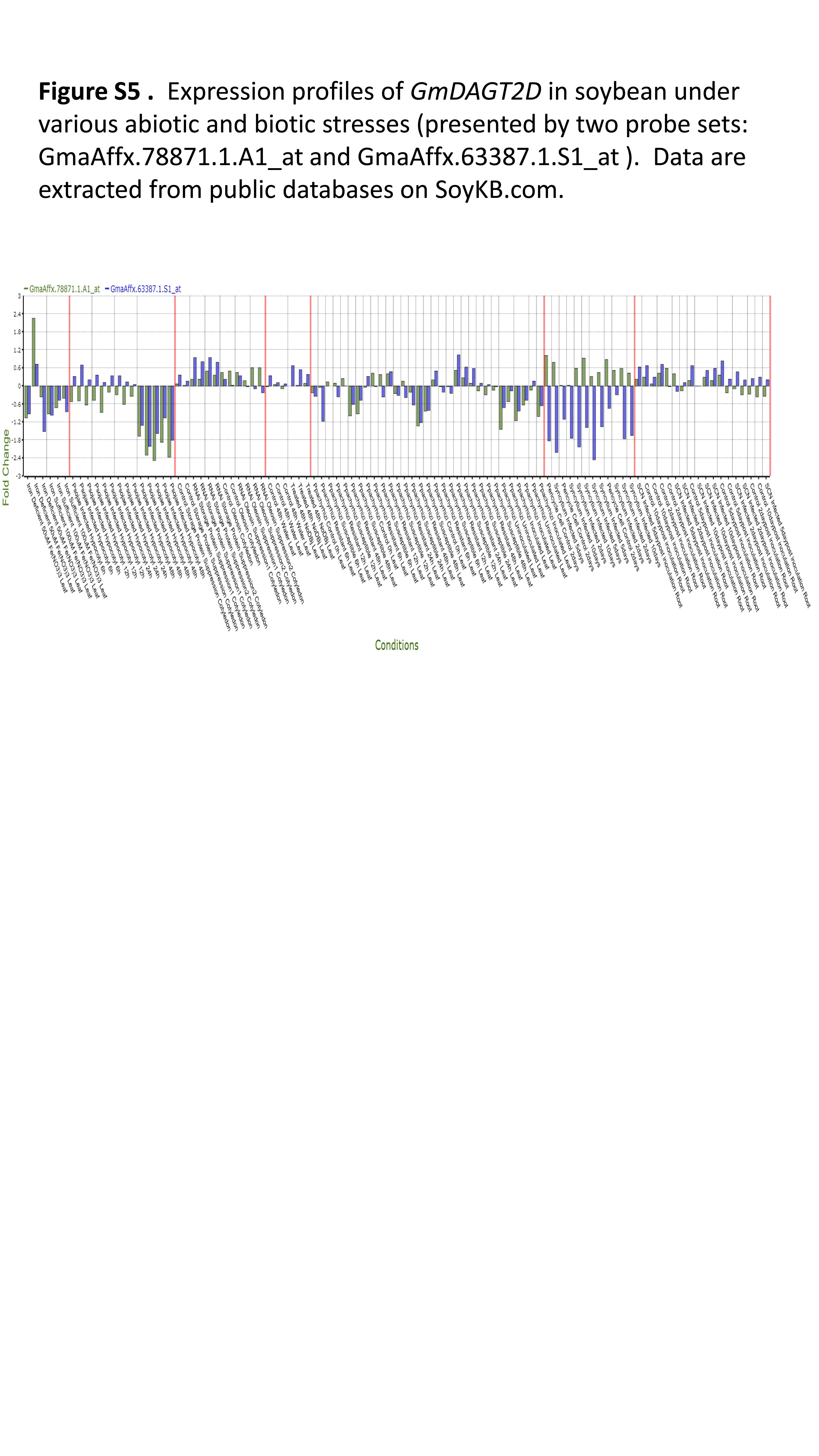


**Supplementary Table S1**. List of primers used in the study

| Primer Names | 5’ - 3’ sequences | Used for |
| --- | --- | --- |
| DGAT2DF | ATGGCGGCGGAACCGGTGAGTG | For cloning cDNA into T-easy vector |
| DGAT2DR | TCAAACTATTCTTAACTC AAGGTTT |
| DGAT1AF | ATGGCGATTTCCGATGAGC |
| DGAT1AR | TCAGTCAAGTTTGCCTTTCCTATTC |
| DGAT2LF | GGGGACAAGTTTGTACAAAAAAGCAGGCTTCATGGCGGCGGAACCGGTGAGTG | For cloning into pDONOR |
| DGAT2LR | GGGGACCACTTTGTACAAGAAAGCTGGGTTCAAACTATTCTTAACTCAAGGTTT |
| DGAT1ALF: | GGGGACAAGTTTGTACAAAAAAGCAGGCTTCATGGCGATTTCCGATGAGC |
| DGAT1ALR: | GGGGACCACTTTGTACAAGAAAGCTGGGTTCAGTCAAGTTTGCCTTTCCTATTC |
| DGAT2DproF | CAATTAAACGCAACACACCACC | Promoter cloning into T-easy |
| DGAT2DproR | GCTC TGACCGCTGAGGGTGT |
| PropDORF | GGGGACAAGTTTGTACAAAAAAGCAGGCTTCCAATTAAACGCAACACACCACC | Promoter cloning into pDONOR |
| DGAT2DPropDORR | GGGGACCACTTTGTACAAGAA AGCTGGGTAGCTCTGACCGCTGAGGGTGT’ |
| 18s univ F | CTATCAACTTTCGATGGTAGG | Testing transgenic Arabidopsis |
| 18s univ R | CCGTGTCAGG ATTGGGTAATTT |
| 35S promoter primer | gacgcacaa tcccactatcc |
| AtACTIN2 Forward | TGGATATCAGGAAGGATCTG | qRT-PCR  At3g18780 |
| AtACTIN2 Reverse | GTGCAACGACCTTAATCTTC |
| DGAT2DqRTF | AGAGCATGGCGGCGGAAC | qRT-PCR |
| DGAT2DqRTR | GACGAATCACC AAATTCTTC |
| DGAT1AQF： | ACTCCATCAGCAGCGACGC |
| DGAT1AQR： | GTGACTCTGCAACTGACGGA |
| GmACTINF | CTTCCCTCAGCACCTTCCAA | qRT-PCR |
| GmACTINR | GGTCCAGCTTT CACACTCCAT |

**Supplementary Table S2.** The *cis*-elements in *GmDGAT2D* promoter regions (1.5 kb upstream of the start codon). Analysis was done by using PLACE program (<http://www.dna.affrc.go.jp/PLACE/>)

| Factor or Site Name | **GmDGAT2D** | **GmDGAT1A** | **cis-**  **element**  **sequences** | **function** |
| --- | --- | --- | --- | --- |
| 10PEHVPSBD | 2 | 1 | TATTCT | related to blue, white or UV-A light; |
| 300CORE | 1 | 1 | TGTAAAG | Binds with P-box binding factor (PBF), a DNA-binding protein of the DOF class of transcription factors |
| ABRELATERD1 | 8 | 2 | ACGTG | ABRE-like sequence required for etiolation-induced expression of erd1 (early responsive to dehydration) in Arabidopsis |
| ACGTATERD1 | 20 | 4 | ACGT | ACGT sequence required for etiolation-induced expression of erd1 (early responsive to dehydration) in Arabidopsis |
| ARR1AT | 20 | 16 | NGATT | AGATT is found in the promoter of rice non-symbiotic haemoglobin-2 (NSHB) gene ARR1; |
| CAATBOX1 | 24 | 18 | CAAT | "CAAT promoter consensus sequence" found in legA gene of pea; Sequences responsible for the tissue specific promoter activity of a pea legumin gene in tobacco. |
| CACTFTPPCA1 | 17 | 24 | YACT | Mesophyll-specific gene expression in the C4 plant Flaveriatrinervia, the promoter of the C4 phosphoenolpyruvate carboxylase gene |
| DOFCOREZM | 20 | 29 | AAAG | Bond by Dof1 and Dof2 transcription factors |
| DPBFCOREDCDC3 | 4 | 2 | ACACNNG | Related to ABA-responsive and embryo-specification elements;ABI5 |
| EBOXBNNAPA | 12 | 0 | CANNTG | Differential combinatorial interactions of cis-acting elements recognized by R2R3-MYB, BZIP, and BHLH factors control light-responsive and tissue-specific activation of phenylpropanoid biosynthesis genes |
| GAREAT | 2 | 0 | TAACAAR | GARE (GA-responsive element); Occurrence of GARE in GA-inducible, GA-responsible, and GA-nonresponsive genes found in Arabidopsis seed germination was 20, 18, and 12%, respectively; see S000181 |
| GT1CONSENSUS | 17 | 27 | GRWAAW | GT-1; light; TATA; TFIIA; TBP; HR; SAR; TMV; leaf; shoot |
| GTGANTG10 | 20 | 8 | GTGA | Functional analysis of cis-elements within the promoter of the tobacco late pollen gene g10 |
| INRNTPSADB | 11 | 4 | YTCANTYY | Photosynthesis nuclear genes generally lack TATA-boxes: a tobacco photosystem I gene responds to light through an initiater |
| MYB2CONSENSUSAT | 1 | 1 | YAACKG | MYB recognition site found in the promoters of the dehydration-responsive gene rd22 and many other genes in Arabidopsis; Y=C/T; K=G/T; See S000177 (MYB2), S000175 (MYBATRD22); MYB; rd22BP1; ABA; leaf; seed; stress; |
| MYCATRD22 | 3 | 0 | CACATG | involved in dehydration; water stress; ABA; myc; leaf; shoot; ICE1: a regulator of cold-induced transcriptome and freezing tolerance |
| MYCCONSENSUSAT | 12 | 0 | CANNTG | MYC; rd22BP1; ABA; leaf; seed; stress; CBF3; cold; CBF/DREB1 |
| PYRIMIDINEBOXHVEPB1 | 1 | 1 | TTTTTTCC | EPB; cysteine proteinase; GA; ABA; GARE; pyrimidine box; seed EPB; cysteine proteinase; GA; ABA; GARE; pyrimidine box; seed; |
| RHERPATEXPA7 | 8 | 1 | KCACGW | Functional Conservation of a Root Hair Cell-Specific cis-Element in Angiosperms with Different Root Hair Distribution Patterns. |
| T/GBOXATPIN2 | 3 | 0 | AACGTG | Involved in jasmonate (JA) induction ; bHLH-Leu zipper JAMYC2 and JAMYC10 proteins specifically recognaize this motif (Boter et al.,2004); T/G-box; JA; pin2; LAP; MYC; wounding |
| WBOXNTERF3 | 1 | 2 | TGACY | transcriptional repressor ERF3 ; May be involved in activation of ERF3 gene by wounding, ERF3; wounding; |
